# Neuropharmacokinetic visualization of regional and subregional unbound antipsychotic drug transport across the blood–brain barrier

**DOI:** 10.1038/s41380-021-01267-y

**Published:** 2021-09-03

**Authors:** Dominika Luptáková, Theodosia Vallianatou, Anna Nilsson, Reza Shariatgorji, Margareta Hammarlund-Udenaes, Irena Loryan, Per E. Andrén

**Affiliations:** 1 Department of Pharmaceutical Biosciences, Medical Mass Spectrometry Imaging, Uppsala University, Uppsala, Sweden; 2 Science for Life Laboratory, Spatial Mass Spectrometry, Uppsala University, Uppsala, Sweden; 3 Department of Pharmacy, Translational PKPD Research Group, Uppsala University, Uppsala, Sweden

**Keywords:** Drug discovery, Biological techniques, Schizophrenia, Neuroscience

## Abstract

Comprehensive determination of the extent of drug transport across the region-specific blood–brain barrier (BBB) is a major challenge in preclinical studies. Multiple approaches are needed to determine the regional free (unbound) drug concentration at which a drug engages with its therapeutic target. We present an approach that merges in vivo and in vitro neuropharmacokinetic investigations with mass spectrometry imaging to quantify and visualize both the extent of unbound drug BBB transport and the post-BBB cerebral distribution of drugs at regional and subregional levels. Direct imaging of the antipsychotic drugs risperidone, clozapine, and olanzapine using this approach enabled differentiation of regional and subregional BBB transport characteristics at 20-µm resolution in small brain regions, which could not be achieved by other means. Our approach allows investigation of heterogeneity in BBB transport and presents new possibilities for molecular psychiatrists by facilitating interpretation of regional target-site exposure results and decision-making.

## Introduction

The unique role of CNS barriers including the blood–brain (BBB) and blood–cerebrospinal fluid (BCSFB) barriers in regulating neural microenvironments and the transport of xenobiotics has been recognized in multiple studies. A growing body of clinical evidence indicates that CNS barriers are involved in both the pathogenesis and the pathophysiology of several mental disorders including schizophrenia (SZ) [1–3]. The changes associated with SZ are complex and range from very fine ultrastructural changes in neurovascular units to clinically documented functional changes in drug transport across the BBB at the level of entire brain regions [4–7]. Recent studies by researchers in the BBB community have highlighted important unanswered questions pertaining to (a) the potential link between antipsychotic medication and impairment of BBB function, (b) targeting of BBB transport mechanisms to improve the effectiveness of antipsychotic medication, and (c) the value of therapeutic strategies aimed at protecting or repairing the BBB in psychosis [2]. Systematic preclinical investigations focusing on the extent of BBB transport of marketed and novel antipsychotics in brain regions of interest (ROI) will be needed to answer these questions. However, the scope for conducting such investigations is limited by the difficulty of quantitatively characterizing the extent of unbound drug delivery to the brain. In preclinical studies, methodological limitations often make it necessary to treat the BBB as a homogeneous interface, implying uniform brain-wide drug exposure. However, in reality, the BBB exhibits considerable structural and functional region-specific heterogeneity [8, 9], which may cause significant errors in the acquisition and interpretation of experimental data if not properly accounted for.

To understand the biological mechanisms of low-molecular-weight drug transport across the BBB, it is essential to recognize that only unbound (free) and nonionized molecules can cross the membrane to interact with targets in the brain and initiate a pharmacological response [10–13]. However, in both blood and brain compartments, drugs exist in both their unbound forms and in bound forms complexed with blood proteins or cell constituents [11]. Specific and sensitive analytical methods are needed to investigate the quantitative impact of multiple interrelated processes on unbound antipsychotic drug transport across the BBB at regional and subregional levels, where subtle differences may exist.

Current methods for investigating the transport of molecules across the BBB include in vivo positron emission tomography brain imaging [14], advanced microscopy [15], in situ brain perfusion [16], and cutting-edge in vitro cell culture models [17–19]. However, in vivo cerebral microdialysis [20] is the only technique that can be used to obtain samples from focal brain areas and blood containing only the unbound drug. This method also permits quantitation of the extent and predominant direction of BBB drug transport based on measurements of *K*_p,uu,brain_, i.e., the ratio of the unbound drug’s concentration in the brain to that in the plasma. Unfortunately, the results of cerebral microdialysis analyses of specific brain regions cannot be directly extrapolated to other regions or the entire brain. Therefore, there is no available method that can provide definitive information on how much unbound drug enters the entire brain or individual brain regions and subregions from the blood at a given time point. However, such detailed knowledge of the pharmacokinetics at different regional target sites within the brain and their interactions is needed to clarify the relationship between unbound drug exposure and observed responses.

Methodological advances in neuropharmacokinetic (neuroPK) studies have enabled assessment of the net flux of substances across the BBB without confounding due to drug binding in the brain and plasma [21, 22]. This was made possible by the development of high-throughput brain-slice method for investigation of drug uptake and distribution into the brain tissue, which is characterized based on the unbound volume of drug distribution in the brain, V_u,brain_. Because V_u,brain_ provides an in vivo-like estimate of the drug’s intra-brain distribution, this approach allows reliable interconversion of estimated total and unbound drug brain concentrations [23, 24]. The brain-slice method is an integral part of the combinatory mapping approach (CMA), a toolbox for assessing intracellular and extracellular exposure of antipsychotics in the entire brain and discrete brain ROI [25]. The CMA was developed for screening and selection of new chemical entities and has expanded the scope of neuroPK studies [25, 26]. Unfortunately, it has a number of technical drawbacks, including low accuracy when applied to small anatomical structures in the brain. This limits the ability to acquire spatial information and makes it impossible to sample subregions or to obtain adequate sample volumes for drug bioanalysis when using conventional liquid chromatography-tandem mass spectrometry (LC–MS/MS).

Matrix-assisted laser desorption/ionization mass spectrometry imaging (MALDI-MSI) enables powerful in situ two-(and three)-dimensional visualization of drugs, drug metabolites, lipids, peptides, and proteins in tissue samples and other matrices [27]. Recent improvements in the lateral resolution, speed, mass resolution, and sensitivity of mass spectrometers, as well as in bioinformatics tools, have enabled visualization of molecules even at subcellular levels [28]. Furthermore, the development of fully validated quantitative MSI (qMSI) methods [29] has allowed MSI techniques to be used in pharmaceutical research also with focus on the assessment of BBB drug transport [30–34]. We recently developed a qMSI approach for investigating how drug–drug interactions influence drug transfer from the blood to small brain structures [35]. This showed that qMSI has applications outside compound mapping and can be used to identify relevant preclinical drug–drug interactions based on binding to specific transporter proteins. However, while existing qMSI methods can accurately determine the distribution of different pharmaceutical entities, they cannot discriminate between bound and unbound drug forms, and therefore can only quantitate total drug concentrations in tissues.

We here present a method, qMSI for unbound drug determination (qMSI-uD), that can be used to assess the extent of unbound drug transport across the BBB and intrabrain drug distribution in small anatomical regions including subregions. By using the power of MALDI-qMSI, we overcome the limitations of the CMA–ROI method and enhance its capabilities. We demonstrate the performance and validity of qMSI-uD using three antipsychotic drugs with different BBB transport properties and regional distribution patterns: risperidone, clozapine, and olanzapine revealing their unique BBB transport features. The method provides a label-free and multiplex approach for detailed mapping and visualization of the extent of regional unbound drug transport across the BBB. Moreover, it enables simultaneous detection and localization of drug metabolites with high lateral resolution. The ability to provide detailed information on unbound antipsychotic drug transport across the BBB and regional target-site exposure makes qMSI-uD a valuable tool for molecular psychiatrists engaged in investigation of various BBB drug transport-related hypotheses.

## Results

### Overview of the concept

**Fig. 1:**
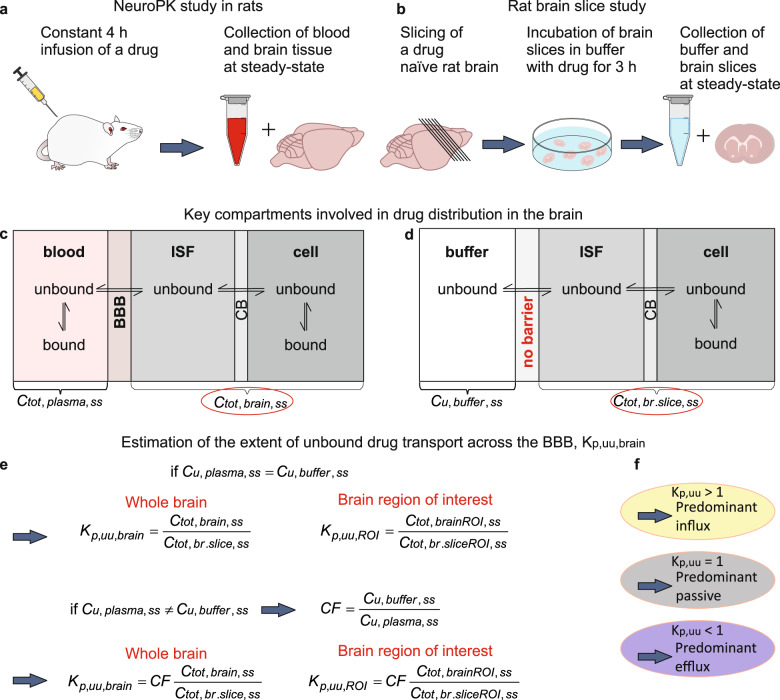
Conceptual overview for mapping the extent of unbound drug transport across the BBB. **a** The in vivo rat neuropharmacokinetic (neuroPK) study provides measurements of the total concentration of a drug in the brain tissue (C_tot,brain,ss_) and blood (C_tot,plasma,ss_) under steady-state conditions achieved by a 4-h continuous intravenous infusion of the drug. **b** The in vitro rat brain-slice method provides measurements of the total concentration of a drug in a brain slice (C_tot,br,slice,ss_) and buffer (C_u,buffer,ss_) under steady-state conditions established by incubating brain slices in the buffer with the drug. **c**, **d** Key compartments when analyzing drug distribution in the brain: blood/buffer, brain interstitial fluid (ISF), and parenchymal cells. **c** Schematic depiction of the in vivo distribution of an unbound drug in the virtually protein-free brain ISF, including passage across the brain endothelial (BBB) and parenchymal cellular (CB) barriers, as well as specific and nonspecific binding to tissue constituents. **d** Under in vitro conditions, distributional processes are analogous, apart from the absence of functional BBB. **e**, **f** Estimation of the extent of unbound drug transport. **e** K_p,uu,brain_ is assessed as the ratio of C_tot,brain,ss_ to C_tot,br,slice,ss_. The underlying assumption is that the steady-state unbound plasma concentration (C_u,plasma,ss_) is equal to the buffer concentration (C_u,buffer,ss_). If this condition is not satisfied, a correction factor (CF) equal to the ratio of C_u,buffer,ss_ to C_u,plasma,ss_ is used. **f** Interpretation of BBB transport based on K_p,uu,brain_ values.

**Fig. 2:**
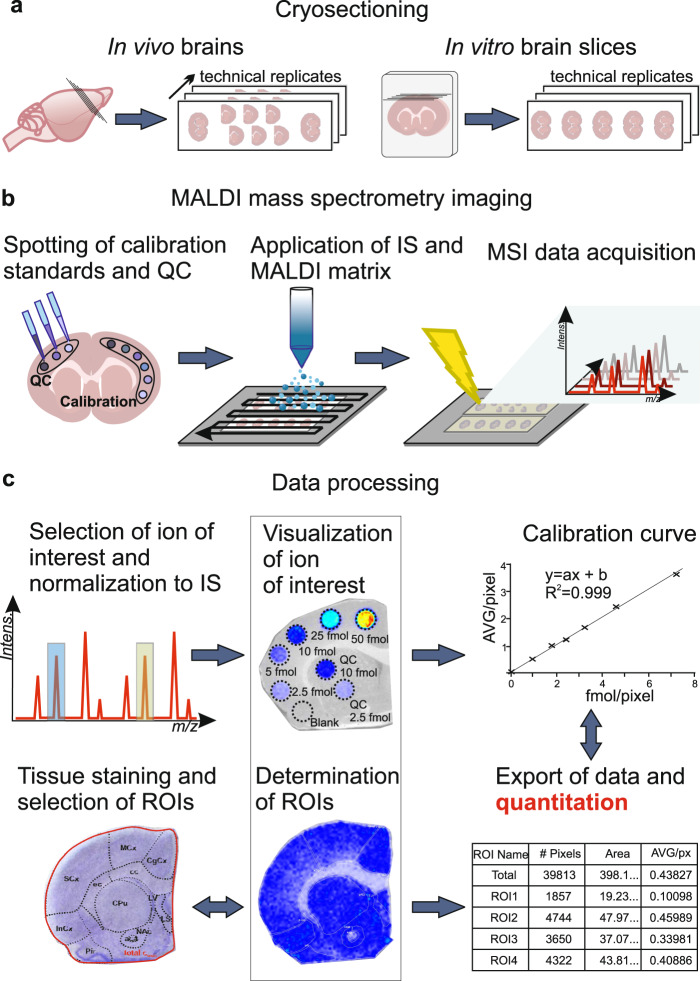
Illustration of the workflow for assessing total brain drug concentrations using qMSI-uD. **a** Cryosectioning of a brain tissue section (left) and a brain slice (right), collected and frozen after performing in vivo and in vitro experiments, respectively. Thaw-mounting of tissue slices on precooled indium tin oxide-coated glass slides and preparation of three technical replicates from consecutive tissue sections on additional glass slides. **b** Sample preparation for MALDI-qMSI. Spotting of calibration standard solutions of the drug and quality-control (QC) solutions on the top of the tissue. Automatic spraying of a deuterated analog of the drug onto the tissue as an internal standard (IS) followed by application of the MALDI matrix and acquisition of MALDI-MSI data. **c** Processing of the acquired data involves selection and visualization of ions of interest and normalization of the data against that for the IS. This is followed by determination of regions of interest (ROI) of calibration-standard spots and selected brain regions based on a reference brain atlas [50] generated using the same tissue sections after staining with hematoxylin and eosin. Finally, a calibration curve is created by plotting the drug’s concentration against the average intensity of the ion, and individual average ion intensities within the region of interest are exported and quantitated using the calibration curve.

Drawing on the pharmacokinetic basis (see Methods), qMSI-uD enables detailed mapping of regional and subregional BBB drug transport based on K_p,uu,brain_ (Fig. 1 and Fig. 2). It combines two distinct experimental approaches, namely in vivo brain neuroPK and in vitro brain-slice drug-distribution studies (Fig. 1). The in vitro rat brain-slice assay provides information on the intrabrain distribution of an unbound drug but cannot provide quantitative information on BBB transport, unlike in vivo data on drug distribution in the brain. The central hypothesis underpinning qMSI-uD is that the ratio of the total brain steady-state concentrations obtained in neuroPK studies (in vivo) and rat brain-slice studies (in vitro) reflects the magnitude and direction of unbound drug transport across the BBB if the unbound drug plasma concentration is equal to the buffer concentration (Eqs. 6 and 7). If the latter condition is not satisfied, i.e., the unbound drug plasma and buffer concentrations are unequal in the steady state, a correction factor must be applied to this ratio (Eq. 9). This may be necessary if the BBB transport and intrabrain distribution of a drug are concentration-independent in a given concentration range. Moreover, using only in vitro rat brain-slice assay data, the intrabrain distribution of an unbound drug can be characterized based on the volume of distribution of unbound drug (V_u,brain_), which can be independently evaluated by determining the ratio of the total concentration of the drug in the brain slice to the steady-state buffer concentration.

The qMSI-uD concept builds on several implicit and explicit assumptions (see Methods) that define the conditions under which it can be used and the validity of inferences drawn from the results obtained. An important and unique advantage of absolute quantitation based on MALDI-qMSI analysis included in the qMSI-uD method (Fig. 2) is that it can provide information on the spatial distribution in the brain of both parent drugs and potentially all of their detectable metabolites.

### Cross-validation of MALDI-qMSI and LC–MS/MS methods for drug bioanalysis, and comparison of their performance to the conventional CMA method

**Fig. 3:**
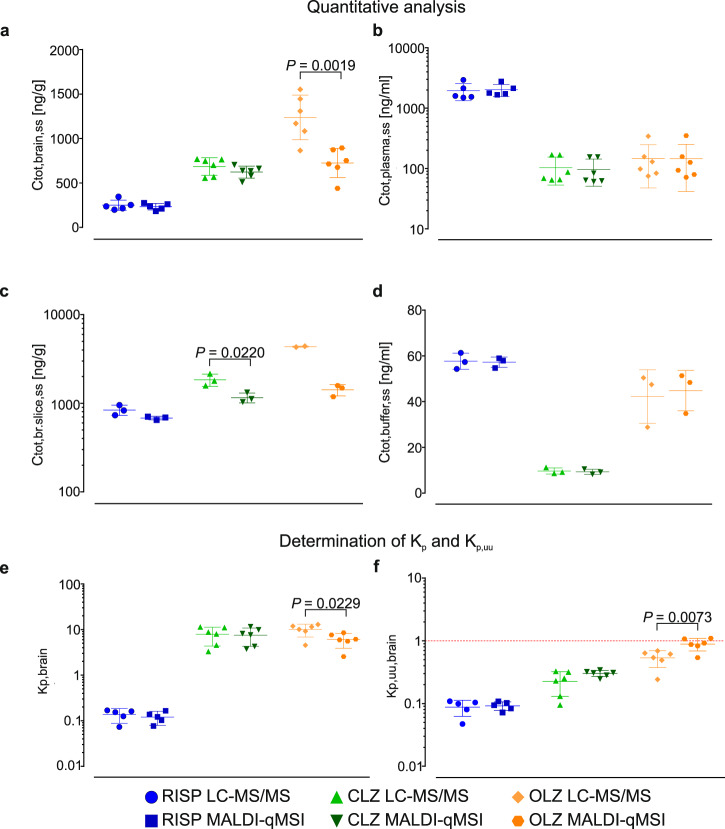
Cross-validation of the LC–MS/MS and qMSI. **a**, **b** Scatter dot plots showing LC–MS/MS and MALDI-qMSI-based quantitation of steady-state total brain tissue and plasma concentrations and **c**, **d** the total brain slice and buffer concentrations collected after performing in vivo experiments (*n* = 6 per group) and in vitro experiments (*n* = 3 per group), respectively. K_p,brain_ (**e**) and K_p,uu,brain_ (**f**) values determined using the conventional CMA method (LC–MS/MS) and MALDI-qMSI. Error bars represent standard deviations from the mean. Total concentrations (C_tot,brain,ss_) for the in vivo samples were analyzed in half of the brain (LC–MS/MS), and in the coronal section of the second half of the brain (MALDI-qMSI). Total concentrations (C_tot,br.slice,ss_) from the in vitro study were analyzed either in the entire brain slice (LC–MS/MS) or in the coronal section of the brain slice (MALDI-qMSI). RISP risperidone, CLZ clozapine, OLZ olanzapine. NB: a semilogarithmic scale is used in panels **b**, **c**, **e**, and **f**.

We benchmarked qMSI-uD against the CMA approach in a sequential manner. First, the neuroPK parameters of the model drugs risperidone, clozapine, and olanzapine were determined at the whole-brain level. To test the validity of our hypothesis (see Eqs. 7 and 9), the newly proposed approach (Eq. 9) and qMSI-uD were compared with conventional CMA (Eq. 5). The *K*_p,uu,brain_ values determined using these three approaches did not differ significantly (Supplementary Fig. 1). Second, the MALDI-qMSI method was validated by applying selected validation criteria for bioanalytical methods based on US FDA guidelines [36] (Supplementary Table 1, 2 and Supplementary Fig. 2). Third, the bioanalysis of the model drugs using MALDI-qMSI was cross-validated against LC–MS/MS data (Fig. 3). There were no significant differences between the two methods for total risperidone concentrations in brain tissues, plasma, and buffer samples. However, the total concentration of clozapine determined by MALDI-qMSI in brain-slice samples was 1.6-fold higher (*P* = 0.022) than that determined by LC–MS/MS (Fig. 3c). A similar but more pronounced pattern was observed for both in vivo (1.7-fold, *p* = 0.002) and in vitro (2.8-fold) brain samples containing olanzapine (Fig. 3a, c). To rule out the possibility that differences in ionization might give rise to differences in analyte quantitation between the two analytical methods, extracts of brain slices dosed with clozapine and olanzapine were analyzed by MALDI-qMSI. The concentrations of clozapine and olanzapine extracts determined using MALDI-qMSI did not differ significantly from those determined by LC–MS/MS (*P* = 0.0508 and *P* = 0.1087, respectively, for the two drugs). The cross-validation results thus suggest that measurements of total drug concentrations in the studied matrices (Fig. 3a–d) by MALDI-qMSI might depend on sample preparation, implying that the results obtained when analyzing whole-brain homogenates may differ from those for coronal brain sections. This would not affect the method’s usefulness for determining neuroPK parameters because the final results of such analyses are expressed as ratios of the values obtained from in vivo and in vitro samples. Importantly, the neuroPK parameters derived using qMSI-uD were similar to the reference CMA values for risperidone and clozapine, and differed from the CMA values by less than a factor of 1.5 for olanzapine (Fig. 3e, f). The method thus achieved good agreement with the conventional CMA approach, supporting its use for region annotation.

### MALDI-qMSI assessment of antipsychotic exposure in rat brain tissue

**Fig. 4:**
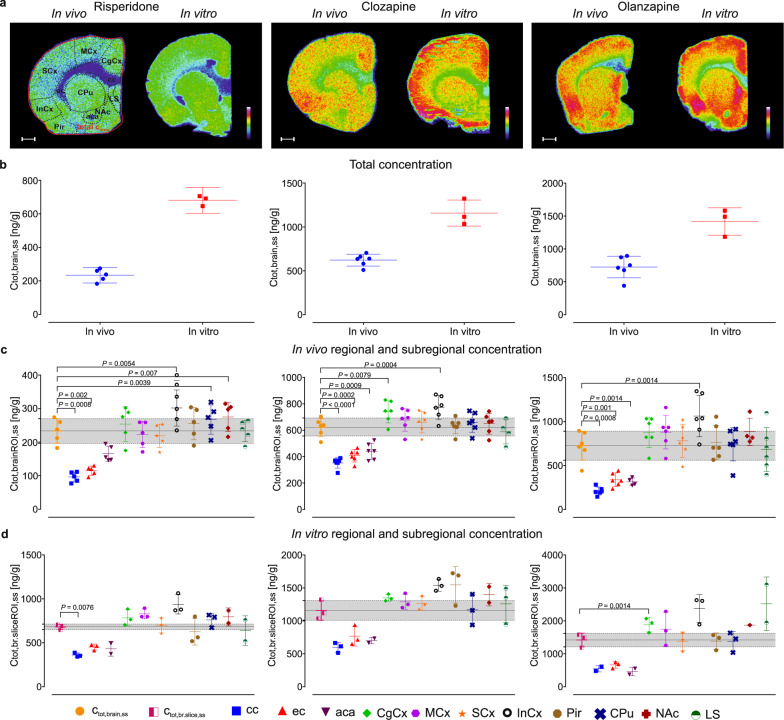
Total brain concentrations determined by MALDI-qMSI. **a** Representative images showing the relative abundance and distribution of risperidone (left), clozapine (middle), and olanzapine (right) in in vivo and in vitro brain tissue sections. Representative images were measured with a lateral resolution of 40 µm and concentrations are shown using a logarithmic scale, scaled to 0–100% (the scale bar is 1 mm). Data for determination of total concentrations were acquired at 100-µm lateral resolution and normalized against a deuterated drug analogue. **b** Scatter dot plots of the total drug concentrations (ng g^−1^) in entire brain sections from in vivo (*n* = 6 per group) and in vitro (*n* = 3 per group) experiments. **c** Scatter dot plots of region-annotated total concentrations obtained from in vivo neuroPK studies, and **d** in vitro rat brain-slice assays. The steady-state total concentrations achieved in vivo (C_tot,brain,ss_) and in vitro (C_tot,br.slice,ss_) are shown as a reference line with the mean ± the standard deviation from the mean (gray shaded area). cc corpus callosum, ec external capsule, aca anterior commissure, CgCx cingulate cortex, MCx motor cortex, SCx somatosensory cortex, InCx insular cortex, Pir piriform cortex, CPu caudate putamen, NAc nucleus accumbens, LS lateral septum. Only statistically significant differences (*P* < 0.01) are shown. Note the differences in scale in figures b–d.

Unbound drug transport across the BBB (Eqs. 7 and 8) was estimated (see Fig. 4) by comparing whole-brain and region-annotated steady-state concentrations of risperidone, clozapine, and olanzapine (Supplementary Table 3) in in vivo and in vitro samples (see Fig. 1). In this context, the term “whole brain concentration” means the concentration in an entire coronal brain section. Mean total drug concentrations measured by MALDI-qMSI in whole-brain sections from in vivo and in vitro experiments (Fig. 4a, b) were used as reference values in Dunnett’s multiple-comparison tests against region-annotated concentrations (Fig. 4c, d and Supplementary Table 4). In vivo exposures in the white matter regions (corpus callosum, external capsule, and anterior commissure) were significantly lower than the total whole-brain concentrations for all three drugs. The lowest concentration was detected in the corpus callosum (Supplementary Table 3). Additionally, the concentrations of risperidone, clozapine, and olanzapine in the insular cortex (a gray matter region) were 1.3-fold (*P* = 0.0054), 1.2-fold (*P* = 0.0004), and 1.5-fold (*P* = 0.0014) higher, respectively, than the corresponding whole-brain concentrations (see Fig. 4c). The ratios of the region-annotated drug concentrations to the whole-brain total concentrations for the in vitro brain-slice experiments exhibited similar trends to those observed in the in vivo neuroPK study (Fig. 4d). Total concentrations do not reflect the extent of unbound drug transport across the BBB but are used to estimate it.

### Mapping of unbound drug transport across the BBB in the brain regions and subregions

**Fig. 5:**
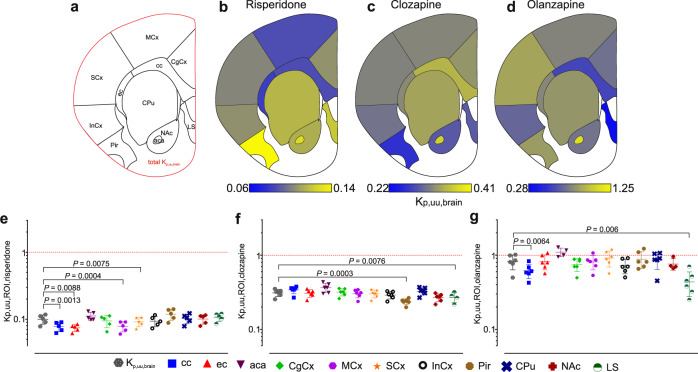
Mapping the extent of unbound drug transport across the BBB on regional and subregional levels using K_p,uu_. **a** Brain map showing the imaged brain section (red line) and annotated brain regions. **b–d** Heat maps of regional K_p,uu,ROI_ values for risperidone, clozapine, and olanzapine (limits are defined by the minimal and maximal mean K_p,uu,ROI_ values). **e–g** Individual scatter dot plots of the regional K_p,uu,ROI_ values. The K_p,uu,brain_ value measured on the level of the entire coronal section is shown as a reference point, and a K_p,uu,ROI_ of unity (indicating predominantly passive BBB transport) is indicated by a red dashed line. Error bars represent the standard deviation of the mean (a semilogarithmic scale is used in the panels). Statistically significant (*P* < 0.01) differences are highlighted. cc corpus callosum, ec external capsule, aca anterior commissure, CgCx cingulate cortex, MCx motor cortex, SCx somatosensory cortex, InCx insular cortex, Pir piriform cortex, CPu caudate putamen, NAc nucleus accumbens, LS lateral septum.

The extent of unbound drug transport across the BBB was estimated based on K_p,uu,brain_ at the whole-brain level and K_p,uu,ROI_ at specific regional levels using Eqs. 7–9. The determined total K_p,uu,brain_ values of 0.10 ± 0.02 for risperidone, 0.31 ± 0.03 for clozapine, and 0.82 ± 0.19 for olanzapine agreed well with previous reports [26]. The spread of these values shows that the three selected drugs span the spectrum of BBB transport behaviors, from active efflux to passive transport. Furthermore, region-annotated mean K_p,uu,ROI_ values obtained by qMSI-uD were used to build unique drug-specific visual maps of BBB drug transport (Fig. 5a–d) that enable at-a-glance scrutiny of BBB transport heterogeneity.

The three drugs had distinct patterns of BBB transport with a few commonalities (Supplementary Tables 5 and 6 and Supplementary Fig. 3). Olanzapine exhibited the most heterogeneous BBB transport, with predominant passive transport and potential uptake in the anterior commissure and somatosensory cortex (Fig. 5d, g). On average, the extent of BBB transport of olanzapine was lowest in the lateral septum: the K_p,uu,ROI_ for this drug in the lateral septum was between 1.4-fold and 2.5-fold lower than in other brain regions (Supplementary Table 5 and 6c). Moreover, the differences in intraindividual region-annotated levels of olanzapine were more pronounced than the differences between individual animals. Unlike olanzapine, the BBB transport of clozapine did not differ greatly between regions or subregions: in all cases, its transport appeared to be dominated by moderate efflux (Fig. 5c, f). Clozapine efflux was most efficient in the piriform cortex with the K_p,uu,ROI_ value differing by less than a factor of 1.5 compared with other regions (Supplementary Table 5 and 6b). Risperidone exhibited strong efflux with moderate differences between the studied regions and subregions (Fig. 5b, e). Interestingly, cortical subregions exhibited greater variation in the extent of BBB transport of risperidone (Supplementary Tables 5 and 6a), with the motor cortex having the most efficient efflux (K_p,uu,ROI_ = 0.08 ± 0.01) and the piriform cortex the least efficient (K_p,uu,ROI_ = 0.12 ± 0.02). Some further common trends were revealed by K_p,uu,ROI_ mapping. The anterior commissure had the highest K_p,uu,ROI_ for clozapine and olanzapine across all discrete regions, and the second highest for risperidone (Supplementary Table 5). Surprisingly, the opposite was observed in another white matter region, the corpus callosum, for which the efflux activity toward risperidone and olanzapine was greater than in other brain regions. These results show that our new method enables reliable estimation of unbound BBB transport in specific regions and subregions of the brain, as well as spatial visualization of intrabrain unbound drug distributions (Supplementary Fig. 5), which cannot be achieved by other means.

### Localization of risperidone and its hydroxylated metabolite using high-resolution imaging

**Fig. 6:**
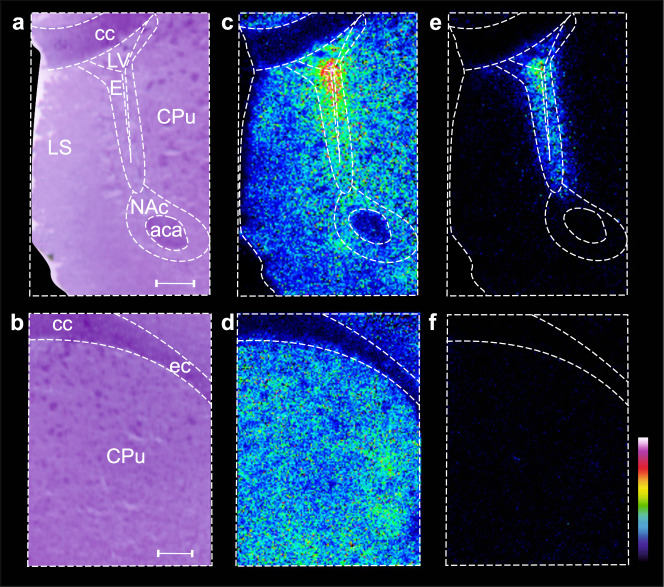
MALDI MS images showing the distribution of risperidone and its metabolite, hydroxyl risperidone, in a representative brain tissue sample with a lateral resolution of 20 µm. **a**, **b** Structural morphology was correlated with hematoxylin and eosin-stained brain tissue after MALDI-MSI analysis. **c**, **d** Ion distribution of risperidone in the brain tissue section. **e**, **f** Ion distribution of hydroxyl risperidone in the brain tissue section. Ion intensities are normalized against that for the ion of the deuterated analog of risperidone; absolute ion intensities are shown using a rainbow color code scaled to a linear scale ranging from 0 to 5. Scale bar: 500 µm. CPu caudate putamen, NAc nucleus accumbens, LS lateral septum, cc corpus callosum, ec external capsule, aca anterior commissure, LV lateral ventricle, E ependymal and subependymal layers of lateral ventricle.

In addition to its capabilities in quantitative drug assessment, MALDI-qMSI could be used for simultaneous detection of drug metabolites and drug-target effects, as well as precise localization of drug metabolites using high-resolution imaging. To illustrate this capability, the distribution of risperidone and its active metabolite hydroxyl risperidone (Fig. 6 and Supplementary Fig. 4) were determined in the striatum and lateral ventricle brain area (interaural 11.16 mm, bregma 2.16 mm). Risperidone and hydroxyl risperidone were most heavily accumulated inside the lateral ventricle, with 1.5-fold lower abundance in the surrounding ependyma (Fig. 6b, c) and the lowest abundance in the dorsal striatum (Fig. 6e, f). The striatal distribution of risperidone was more pronounced in the gray matter of the striatum than in neuronal fibers. Whereas the abundance of risperidone was the highest in the anterior commissure and lowest in the corpus callosum, the opposite was observed for hydroxyl risperidone. Coupling qMSI-uD with high-resolution imaging will enhance the method’s capabilities, providing insights into BBB transport at the intraregional level. To illustrate the advantages of the MALDI-MSI methodology in measuring drug-target effects using the same brain tissue material, we provide an example of the impact of risperidone on the dopaminergic system (Supplementary Fig. 6). The ability to quantify regional unbound drug exposure within the brain and simultaneously evaluate target-interaction effects enables a better understanding of the complex processes that regulate a drug’s concentration profile within the brain at the target site. The latter ability in particular presents new opportunities for molecular psychiatrists to clarify pharmacokinetic–pharmacodynamic relationships at the level of individual brain regions.

## Discussion

We have developed a method, qMSI-uD, that enables simultaneous determination and visualization of the extent of unbound pharmacologically active antipsychotic drug transport across the BBB (K_p,uu,brain_) in small anatomical regions of the brain using MALDI-qMSI technology. The combination of two complementary experimental approaches that involve establishing pharmacokinetic equilibrium, namely in vivo neuroPK and in vitro brain-slice studies, allows the extent of unbound BBB transport of small-molecular-weight substances to be estimated using Eqs. 7 or 9. The resulting regional (K_p,uu,ROI_) values are subsequently used to generate region-annotated maps showing the local extent and predominant direction of unbound drug transport across the BBB. In addition, qMSI-uD enables spatial visualization of intrabrain unbound drug distributions, which depend on specific and nonspecific drug brain tissue binding, pH partitioning, and cellular barrier transport. These distributions are characterized using the variable V_u,brain_ [24], which can be used to estimate regional unbound drug concentrations in the interstitial fluid based on total concentrations obtained from in vivo neuroPK studies. The ability to simultaneously characterize both the extent of regional BBB drug transport and the post-BBB regional distribution of unbound drugs in the brain with high spatial resolution offers neuroscientists unprecedented opportunities to characterize, rank, and pharmacologically profile substances based on their neuroPK properties in specific target regions of the brain. The proposed approach could also potentially be applied to nonbrain tissues, further extending its applicability.

The BBB is a highly specialized and selective interface consisting of brain microvascular endothelial cells with tight junctions surrounded by an intricate milieu of astrocyte end feet and pericytes [37]. Its microvasculature topology, neurovascular cellular composition, and endothelial biochemical characteristics are all region-specific and heterogeneous [8, 9, 38]. Endothelial dysfunctions in the BBB are widely accepted to play central roles in the development and progression of several neurological diseases including SZ [39]. Abnormalities of capillaries and their microenvironment in the prefrontal and visual cortices in SZ brains include thickening of the basal lamina, prominent swelling of astrocytic end feet, and signs of microglial activation [6]. Molecular alterations in brain endothelial cells in the prefrontal cortex in SZ patients include elevated levels of intercellular adhesion molecule-1 and VE-cadherin mRNA, and exacerbated reductions in the levels of breast cancer resistance protein in the “high inflammation” SZ subgroup [7]. In addition, positron emission tomography experiments showed that patients with chronic SZ under treatment with antipsychotics exhibited significantly reduced 11C-verapamil uptake at the brain regional level. This was probably related to an increase in the activity of the P-glycoprotein (P-gp) pump [4]. Despite the growing evidence of structural and functional heterogeneity in the BBB and its role in mental disorders, most existing analytical methods cannot be used to study functional aspects of BBB drug transport because they are only capable of determining total drug concentrations. This limitation is overcome by qMSI-uD, which can be used to probe the BBB transport of any substance of interest; it is not limited to drugs and drug candidates.

Using qMSI-uD we demonstrated differences in the extent of BBB drug transport within the gray and white matter for three antipsychotics: risperidone, clozapine, and olanzapine. These drugs exhibited a wide range of BBB transport properties, including both efficient efflux and active uptake. The heterogeneity of the BBB has previously been shown to play a key role in the regional transport of unbound antipsychotics.

The MALDI-qMSI method enabled visualization of regional and subregional differences within coronal rat brain tissue sections for risperidone, clozapine, and olanzapine after drug exposure in vivo and in vitro. The concentrations of the drugs in white matter were found to be up to three times lower than in gray matter. The total concentration of a drug in a given region of the brain, i.e., the sum of the unbound and bound drug concentrations (the latter of which depends strongly on blood exposure), is closely associated with the restrictive function and heterogeneity of the BBB [8], the spatial expression of transporters and receptors [38], and specific and nonspecific drug binding in the brain parenchyma [25, 40]. The lack of a functional BBB in the brain-slice preparation only allows drug uptake and binding to brain tissue to be evaluated. This is why the measured drug exposure in the in vitro brain slices was higher than in the in vivo brain tissue samples.

Previous studies [25, 26] have shown that the *K*_p,uu,brain_ is a key parameter for measurement of drug BBB transport because it reflects the brain availability of the pharmacologically active unbound drug. The value of K_p,uu,brain_ also indicates the dominant process by which the unbound drug passes across the BBB: if K_p,uu,brain_ < 1, efflux dominates; if K_p,uu,brain_ = 1, passive transport dominates; and if K_p,uu,brain_ > 1, the drug is actively taken up. Previously reported K_p,uu,brain_ values for risperidone, clozapine, and olanzapine agree well with our results, differing by less than a factor of two [26]. Regional differences in K_p,uu,ROI_ have been demonstrated—specifically, the value for the frontal cortex was reported to be higher than that for the caudate putamen brain region in the cases of risperidone, clozapine, and olanzapine [25]. The caudate putamen K_p,uu,ROI_ values obtained in this work agree with those reported previously [25], but the K_p,uu,ROI_ values for individual cortex subregions were similar to those for the caudate putamen. These contrasting results could be due to differences in experimental design: the doses of risperidone and olanzapine used in this work were ca. 10- and 3-fold higher, respectively, than those used in the previous study, which may have caused saturation of BBB transporters in this work. Differences in tissue sampling and sample-preparation procedures may also have contributed to the difference in results: whereas the previous study examined homogenates of the entire frontal cortex, we examined coronal sections and divided the cortex into individual subregions. Furthermore, drug transport across the BBB can be highly region-dependent due to the heterogeneity of brain microvasculature [41] and the variation in the density of transporters and receptors along the BBB [38]. In addition, the relatively high total concentrations of risperidone and its metabolite observed in the lateral ventricle could be due to structural, biochemical, and functional differences between the BBB and BCSFB (Fig. 6) [42]. A similar distribution was previously observed by MSI for loperamide, another known P-gp substrate [35].

BBB drug transport is driven by several processes and depends on the number of functional transporters that exist per unit of capillary surface area. However, transporter density differs between brain regions and subregions [9, 43, 44], and the degree of bifurcation, vascular density, and vessel length all differ between cortical subregions [41]. For example, the corpus callosum, external capsule, and lateral septum regions have some of the lowest vascular densities and vessel lengths among mouse brain regions, which may influence the number of available transporters at the BBB per unit surface area [9, 41]. We found that the BBB efflux of clozapine and olanzapine was appreciably stronger in the lateral septum than in other brain regions, while the highest efflux activity for risperidone and olanzapine was in the corpus callosum subregion. It should also be noted that although the total drug concentrations in the corpus callosum were low, this region had some of the highest K_p,uu,ROI_ values observed in this work. Conversely, the highly vascularized piriform cortex had the lowest net efflux of P-gp substrate risperidone at the BBB. The latter could be related to the local expression of efflux transporters, yet the lack of correlation between the expression of the P-gp transporter and its activity has been also documented [45]. Interestingly, the anterior commissure had some of the highest K_p,uu,ROI_ values for all of the tested drugs. The role of regional expression of BBB drug transporters becomes obvious also in relation to the pathophysiology of SZ as well as development of the resistance or increased sensitivity to antipsychotic treatment [4, 5, 46]. At present, there is very little quantitative data on the regional and subregional expression of transporters along the capillaries in normal and SZ brain, as well as in the course of antipsychotic treatment [7, 37, 38, 47]. Data on regional transporter expression could help explain regional differences in the BBB transport of unbound drugs.

Because drug transport across the BBB is so sensitive to its heterogeneity in different brain regions, there is a clear need for robust advanced imaging methods that can reveal the spatial distribution of drugs and their metabolites in the brain. To implement qMSI-uD, it is necessary to satisfy some pharmacokinetic and qMSI-related criteria (as discussed in the Methods section). In particular, it is essential to establish pharmacokinetic equilibrium under all experimental conditions and to optimize the administered doses and concentrations of each drug. Additionally, qMSI requires that all prospective analytes be ionizable with sufficient sensitivity by MALDI-qMSI.

In conclusion, we have developed a method for detailed spatial assessment of key pharmacokinetic parameters (K_p,uu,brain_, V_u,brain_) of antipsychotic drugs in the brain based on the hypothesis that the ratio of the total brain concentrations obtained by in vivo and in vitro neuroPK measurements reflects the extent of BBB transport of an unbound drug. This method can accurately discriminate between a parent drug and its metabolites in a single analysis. Moreover, it provides region-specific data on drug exposure that can be linked to drug-response data, facilitating the discovery of exposure-response relationships for the specific regional target sites as well as decision-making concerning the development of new drug candidates. Although we focus on the BBB here, our method could in principle be applied to any tissue containing a functional barrier. It could also deepen our understanding of existing drug candidates, further increasing its value in molecular psychiatry research.

## Methods

Bioanalysis of samples using reversed-phase LC–MS/MS and MALDI-MSI methods was performed according to general guidelines from American authorities’ requirements on the validation of bioanalytical methods [36] where applicable. Animal studies are reported in accordance with the ARRIVE (Animal Research: Reporting of In Vivo Experiments) guidelines [48].

### Chemicals

Clozapine, olanzapine, clozapine-*d*_*4*_, risperidone-*d*_*4*_, olanzapine-*d*_*8*_, 2-hydroxypropyl-β-cyclodextrin (HPβCD), 4-(2-hydroxyethyl)-1-piperazineethanesulfonic acid (HEPES), dimethyl sulfoxide (DMSO), isopentane, and gelatin were obtained from Sigma-Aldrich (Stockholm, Sweden). Risperidone, acetonitrile, formic acid (FA), trifluoroacetic acid (TFA), 2,5-dihydroxybenzoic acid, cresyl violet, hematoxylin, and eosin were purchased from Merck (Darmstadt, Germany). Absolute ethanol and chloroform were purchased from VWR (Stockholm, Sweden). Water used in all experiments was either HPLC grade (Merck, Darmstadt, Germany) or purified using a Milli-Q Academic system (Millipore, Bedford, MA, USA).

### Animals

All experiments were performed on drug-naive male 250-300 g Sprague–Dawley rats (Taconic, Lille Skensved, Denmark) in accordance with the guidelines of the National Board for Laboratory Animals, and were approved by the Animal Ethics Committee of Uppsala, Sweden (ethical approvals C189/14). All rats were housed in groups at 20–22 °C and 45–65% humidity, under a 12-h light/dark cycle with *ad libitum* access to food and water. All neuroPK experiments were performed after one week of acclimatization.

### In vivo neuroPK studies

The plasma and CNS exposure of the three antipsychotic drugs risperidone, clozapine, and olanzapine were assessed in vivo under steady-state conditions. The minimally required pergroup sample size for a two-tailed t-test study was estimated to be six, given the probability level (*α* = 0.05), the anticipated effect size (Cohen’s *d* = 1.5), and the desired statistical power level (0.8) [49]. Hence, 24 drug-naive rats were included in in vivo neuroPK studies, i.e., six rats per drug or vehicle control. Animals were not randomized, and the study was not blinded.

The optimal dose for each drug was determined based on prior knowledge [25] and pilot experiments (*n* = 1 rat per drug, data not shown). A total plasma concentration of 100 ng mL^−1^ was targeted during optimization of the total brain concentration under steady-state conditions. Due to the comparatively low sensitivity of MSI detection of olanzapine and risperidone, the doses of these drugs were increased. The final total doses were 7.7, 2.5, and 5 mg kg^−1^ for risperidone, clozapine, and olanzapine, respectively.

Each rat’s femoral vein and artery was surgically catheterized one day before the experiment under isoflurane anesthesia. After that, they were individually placed in a CMA120 system (CMA, Solna, Sweden) for freely moving animals with *ad libitum* access to food and water. The drugs were administered individually in 10% hydroxypropyl beta-cyclodextrin in saline for parenteral use (Uppsala University Hospital Pharmacy, Uppsala, Sweden) as a fast-rate infusion for 5 min followed by a 4-h constant slow-rate intravenous infusion, using a flow rate of 1 mL kg h^−1^. One group (*n* = 6 rats) was used as vehicle controls and received 10% hydroxypropyl beta-cyclodextrin in saline for 4 h, using the flow rate applied during drug administration. Blood samples (300 µL) were taken from *a. femoralis* before the start of the infusion and at its termination. Plasma samples were obtained after centrifugation at 4 °C for 5 min at 10,000 rpm and were stored at −80 °C pending bioanalysis. At the end of the experiment, the rats were anesthetized by inhalation of 5% isoflurane (Abbot Scandinavia, Solna, Sweden) for at most three min and decapitated. The brains were rapidly removed, cut sagittally to separate the right and left hemispheres, and immersed in isopentane that had been prechilled on dry ice for 10 s. Subsequently, the brain samples were covered with aluminum foil and placed on dry ice. Samples were stored at −80 °C pending bioanalysis by LC–MS/MS and MSI.

### In vitro brain-slice assay

The unbound volumes of distribution of the antipsychotic drugs in brain (V_u,brain_, mL g brain^−1^) were estimated using the brain-slice method according to previously published protocols [23, 24] with modifications described below. V_u,brain_ reflects the extent of drug binding and uptake into brain parenchymal cells.

To evaluate the possibility of using brain slices after incubation for further cryosectioning as required for MSI analysis, several pilot experiments were performed to identify a suitable slice thickness and duration of incubation to enable equilibration of the drug between the brain-slice interstitial fluid and the buffer in the beaker without reducing the integrity or quality of the slices. In the optimized procedure, five 500-µm brain coronal slices (from the area of rostral striatum) were obtained from each drug-naive rat brain (*n* = 3 per group) using a vibrating blade microtome Leica VT1200 (Leica Microsystems AB, Sweden). The brain slices were then transferred into one Ø80-mm flat-bottom glass beaker containing 15 mL of preoxygenated artificial extracellular fluid (aECF) containing the drug of interest at a final concentration of 200 nM. The slices were incubated for 3 h at 37 °C in a shaker (MaxQ4450 Thermo Fisher Scientific, NinoLab, Sweden) with a rotation speed of 45 rpm and a constant oxygen flow of about 75–80 mL per min through a glass frit. The beaker was covered with a custom-fabricated lid fitted with a Teflon fluorinated ethylene–propylene film (Teflon FEP film 50 Å, 12.7-µm thickness) to allow oxygen exchange and prevent evaporation of the aECF. The pH of the aECF was measured immediately after the 3-h incubation to ensure that it remained within the acceptable range (7.4 ± 0.15). This criterion was satisfied in all experiments. Brain slices from three other rats were incubated in blank aECF under otherwise identical conditions to obtain control brain tissue samples for the MALDI-qMSI analysis. Brain slices and aECF were sampled at the end of the 3-h incubation. The recovery and thermostability of the investigated drugs were assessed by sampling the aECF buffer before and after the 3 h incubation. The recovery and thermostability of all three drugs exceeded the acceptable minimum value of 70% (data not shown). All aECF samples were mixed with blank brain homogenate in a ratio of 1:4 (w:v) and stored at −80 °C pending bioanalysis by LC–MS/MS and MALDI-qMSI. One brain slice from each experiment was sampled for LC–MS/MS analysis. The slices were individually weighed and homogenized in aECF (1:9, w:v) using a VCX-130 ultrasonic processor (Sonics, Chemical Instruments AB, Sweden) and stored at −80 °C pending bioanalysis. The four remaining brain slices from each experiment were used for MALDI-qMSI analysis. The following handling procedure was applied to ensure preparation of high-quality slices suitable for subsequent cryosectioning. First, brain slices were individually transferred to parafilm with a minimal volume of aECF on a spatula and delicately flattened using a brush. Second, the excess buffer was aspirated by gently applying vacuum on the edges of the slice. Thereafter, the flattened brain slice on parafilm was placed on a metal plate that had been prechilled on dry ice and covered with 0.6 mL of ice-cold isopentane. The frozen slices were then immediately placed on dry ice and stored at −80 °C pending MALDI-qMSI analysis.

The concentrations of the drugs in the virtually protein-free aECF at equilibrium were assumed to be equal to their concentration in the interstitial fluid of the brain slices. Therefore, V_u,brain_ (mL g brain^−1^) was estimated using Eq. 1 as the ratio of the amount of compound in the brain slice (A_brain_, ng g brain^−1^) to the measured final unbound concentration of the drug in the aECF (C_u,buffer_, ng mL^−1^). The brain tissue density was assumed to be 1 g mL^−1^.

1 $${{{{{{{\mathrm{V}}}}}}}}_{{{{{{{{\mathrm{u,brain}}}}}}}}}{{{{{{{\mathrm{ = }}}}}}}}\frac{{{{{{{{{\mathrm{A}}}}}}}}_{{{{{{{{\mathrm{brain,slice,ss}}}}}}}}} - {{{{{{{\mathrm{V}}}}}}}}_{{{{{{{\mathrm{i}}}}}}}} \cdot {{{{{{{\mathrm{C}}}}}}}}_{{{{{{{{\mathrm{u,buffer,ss}}}}}}}}}}}{{{{{{{{{\mathrm{C}}}}}}}}_{{{{{{{{\mathrm{u,buffer,ss}}}}}}}}} \cdot {{{{{{{\mathrm{(1}}}}}}}} - {{{{{{{\mathrm{V}}}}}}}}_{{{{{{{\mathrm{i}}}}}}}}{{{{{{{\mathrm{)}}}}}}}}}}$$

Here, V_i_ (mL g brain^−1^) is the volume of the aECF layer surrounding the brain slices, and was estimated to be 0.094 mL g brain^−1^ using [^14^C] inulin as a marker [23]. NB: the impact of the microvasculature on the measurement of the overall uptake of a drug by brain parenchymal cells is negligible. The mean drug concentrations in the buffer after incubating the brain slices for 5 h were on average twice the mean unbound brain interstitial fluid concentrations in vivo. This rules out any potential concentration-dependent differences in intrabrain drug distribution between the in vivo and in vitro settings.

Since the process of preparing the brain slices for MALDI-qMSI analysis eliminated the possibility that the buffer layer could quantitatively affect the estimation of the total drug concentration in the brain slices, Eq. 2 was used to calculate V_u,brain_.

2 $${{{{{{{\mathrm{V}}}}}}}}_{{{{{{{{\mathrm{u,brain}}}}}}}}}{{{{{{{\mathrm{ = }}}}}}}}\frac{{{{{{{{{\mathrm{C}}}}}}}}_{{{{{{{{\mathrm{tot,brain,slice,ss}}}}}}}}}}}{{{{{{{{{\mathrm{C}}}}}}}}_{{{{{{{{\mathrm{u,buffer,ss}}}}}}}}}}}$$

Here, C_tot,brain,slice,ss_ (ng g brain^-1^) is the total amount of the drug in the coronal brain slice measured using MALDI-qMSI. High V_u,brain_ values indicate extensive drug brain tissue binding and/or uptake.

### MALDI-qMSI sample preparation

The MALDI-qMSI-based sample analysis is illustrated in Fig. 2. The frozen brain tissues and brain slices were cryo-sectioned using a Leica CM1900 UV cryostat–microtome (Leica Microsystems, Wetzlar, Germany). After conditioning of the samples in the cryochamber at −20 °C for 1 h, consecutive 12-µm-thick coronal brain sections of the brain tissues were cut at the striatal level and thaw-mounted on a precooled conductive indium tin oxide-coated glass slide (Bruker Daltonics, Bremen, Germany). Brain tissues from the in vivo studies were mounted next to one another on the same slide to minimize variation due to matrix application and MSI data acquisition. The same approach was used for brain tissue slices originating from the in vitro experiments. Three sequential slides for each sample set (in vivo and in vitro) were prepared for use as technical replicates. To enable cryosectioning of the 500-µm brain tissue slices, they were mounted in icy water on a flat gelatin block and frozen prior to sectioning. The prepared slides were stored at −80 °C.

On the day of measurement, the tissue sections were vacuum-desiccated for 40 min prior to application of the calibration standards and quality-control samples, after which they were optically imaged with a photo scanner (Epson Perfection V500). To quantify the drug in the tissue, calibration solutions (40 nL of 25, 50, 100, 250, 500, and 1000 ng mL^−1^) and quality-control samples were spotted on the top of the brain cortex and/or striatum regions of the control tissues. These solutions contained low (25 ng mL^−1^), intermediate (100 ng mL^−1^ for risperidone or 250 ng mL^−1^ for clozapine and olanzapine), or high concentrations (500 ng mL^−1^ for risperidone or 1000 ng mL^−1^ for clozapine and olanzapine) of the appropriate drug in 50% ethanol with 0.2% TFA and were applied using a BioSpot BT600 automatic spotter (BioFluidix, Freiburg im Breisgau, Germany).

Solutions of risperidone-*d*_4_ (30 ng mL^−1^), olanzapine-*d*_8_ (40 ng mL^−1^), and clozapine-*d*_4_ (55 ng mL^−1^), all prepared in 50% acetonitrile with 0.2% TFA, were used as internal standards and were sprayed on top of the tissue sections with an automatic TM sprayer (HTX-Technologies LLC, Chapel Hill, NC, USA) prior to application of the MALDI matrix. The concentration of the internal standard was chosen to match the average signal intensity of the corresponding drug in the relevant brain tissue sections. The internal standard solution was sprayed over the samples under the following conditions: the nozzle temperature of the spray head was set at 90 °C and the reagents were sprayed pneumatically (6-psi N_2_) with a flow rate of 70 µL min^−1^ and a linear nozzle velocity of 110 cm min^−1^ in six passes (alternating horizontal and vertical deposition) with 2-mm track spacing. Subsequently, 2,5-dihydroxybenzoic acid MALDI matrix (35 mg mL^−1^ in 50% acetonitrile with 0.2% TFA) was sprayed with a spray head temperature of 95 °C and a linear nozzle velocity of 120 cm min^−1^ in eight passes with 3 mm track spacing. For high-resolution imaging, the matrix solutions were prepared in 60% acetonitrile with 0.2% TFA.

Drug concentrations in plasma and buffer samples were also measured using the LC–MS/MS protocol described below. The supernatants (10 µL) of extracted plasma and buffer samples, as well as calibration standards and quality-control samples, were diluted with 20 µL of 0.2% TFA and vigorously vortexed. Afterward, 0.5 µL of each sample was spotted manually on a MALDI ground steel plate in triplicate. The supernatants of risperidone plasma samples were diluted tenfold to ensure that their drug concentrations were below the relevant upper limit of quantification (ULOQ), i.e., below 1000 ng mL^−1^. The prepared MALDI plate was then sprayed with 2,5-dihydroxybenzoic acid (35 mg mL^−1^ in 50% acetonitrile with 0.2% TFA) using the TM sprayer with the same application method as for the tissue samples. Internal standards of all drugs were included in the extraction procedure (see LC–MS/MS section). Therefore, there was no need for additional internal standard spraying.

### MALDI-qMSI analysis and data processing

All MSI experiments were performed using a MALDI Fourier-transform ion cyclotron resonance (FTICR) mass spectrometer (solariX 7-T 2Ω, Bruker Daltonics, Bremen, Germany) equipped with a Smartbeam II 2-kHz laser and operated in positive-ion mode. The operating conditions (ion transfer, laser power, and laser focus) were adjusted separately for each measured drug to maximize the signal of the relevant parent ion before the first data acquisition and then kept constant throughout the analysis of the complete sample set. All data were acquired in continuous accumulation of selected ion (CASI) mode with a 160-Da mass window in the mass range 150–600 *m/z* (CASI 340–500 *m/z*) for risperidone and 100–600 *m/z* (CASI 220–380 *m/z*) for olanzapine and clozapine, with 100 laser shots per sample position. Brain tissue (*n* = 6 dosed, *n* = 1 control) and brain slice (*n* = 3 drug-treated, *n* = 1 control) sections were analyzed in one day and in random order to prevent possible bias due to potential factors such as matrix degradation or variation in mass spectrometer sensitivity. All samples were measured in three technical replicates on three consecutive days. Triplicates of plasma samples of all drugs were analyzed on the same day, but triplicates of buffer samples were analyzed on different days. Tissue data were acquired with 100- or 40-µm raster steps. For plasma and buffer data acquisition, a 150-µm raster step was used. High-resolution images were acquired with a 20-µm lateral resolution. The acquired data were normalized against the data for the relevant deuterated analog and visualized using flexImaging software (Bruker Daltonics, v.5.0). Quantitative assessment of the tissue data was accomplished using msIQuant software (v.2.1.1.20), taking into account the density of the rat brain tissue (1.027 g cm^−3^) and the thickness of the sections (12 µm), after converting the flexImaging data to msIQuant format using the msIQuant convertor tool (FI 402 Reader to msIQuant). The results for specific brain regions were extracted after drawing the regions manually based on a rat brain atlas [50]. For olanzapine, clozapine, risperidone, and their deuterated analogs, *m/z* values were extracted using windows of 1 mDa and 1.5 mDa, respectively. MALDI-qMSI data for plasma and buffer samples were evaluated using the SCiLS software package (v.2020a Pro, Bruker Daltonics). For each analyte, the average ion intensity within the ROI (either the whole tissue section or a specific brain region) was used for data exploration. For the plasma and buffer matrices, areas under the curve were used instead of maximum intensities. This option was chosen after loading the raw data into SCiLS. To generate calibration curves, the calibration standards, quality-control samples, and blanks were measured three times: before, in between, and after the acquisition of brain tissue samples. The same approach was used for plasma and buffer samples.

### Morphological evaluation for MALDI-MSI

After MALDI-MSI analyses, tissue sections were subjected to either Nissl staining or chloroform wash. Briefly, Nissl staining was performed by quickly washing with absolute ethanol to remove the MALDI matrix. The tissues were then fixed with two absolute ethanol washes of 5 min each and allowed to dry for another 5 min. After a subsequent one-min wash in water, tissues were stained with cresyl violet acidic solution (0.25% of acetic acid in water) for 10 min. Excess stain was removed by washing for 5 min in water, 3 min each in 90% and 95% ethanol, and then 3 + 3 min in absolute ethanol. For chloroform washing, the slide was placed in anhydrous chloroform for 30 s after MALDI matrix removal and then left to dry at room temperature. Images of stained or chloroform-washed tissues were overlaid on the MS images to facilitate annotation of brain regions. After high-resolution imaging, the tissues were stained with hematoxylin and eosin as follows. The MALDI matrix was removed and the tissues were washed with 95% ethanol for 30 s, 70% ethanol for 30 s, and deionized water for 2 min. Then, the tissues were stained with hematoxylin for 3 min. Excess stain was removed by washing in running tap water for 10 min, after which counterstaining with 1.5% eosin solution in 70% ethanol was performed for 30 s. The final removal of excess stain was performed by washing for 30 + 30 s in 70% ethanol, 30 s in 95% ethanol, and 5 min in absolute ethanol.

### LC–MS/MS sample preparation

Acetonitrile-mediated protein precipitation (water: acetonitrile, 1:3, v:v) was performed for all matrices, i.e., brain homogenate (1:9 in aECF) and plasma. Standards and quality-control samples were then prepared in each matrix using the cassette approach: all samples to be analyzed in the same matrix were pooled and processed as a single sample. A suitable dynamic range was attained for all analytes. The concentration range, based on at least six standard concentrations in the standard curves, was 10–1000 ng mL^−1^ in plasma and 5–1000 ng g^−1^ for brain homogenates. Quality-control samples with low (50 ng mL^−1^ and 5 ng g^−1^), intermediate (200 ng mL^−1^ and 100 ng g^−1^), and high (1000 ng mL^−1^ and 1000 ng g^−1^) levels were prepared in plasma and brain matrices, respectively.

Briefly, 25 µL of samples, blanks, quality controls, and standards were precipitated using 75 µL of ice-cold 0.2% FA in acetonitrile containing 50 ng mL^−1^ of olanzapine- *d*_*8*_, 50 ng mL^−1^ of clozapine- *d*_*4*_, and 10 ng mL^−1^ of risperidone-*d*_*4*_. The precipitated samples were vigorously vortexed and centrifuged for 3 min at 13,000 rpm. Thereafter, 25 µL of the supernatant was diluted with 200 µL of a mixture of 0.1% FA as elution solvent A (MPA). For plasma samples obtained after in vivo administration of risperidone, the supernatant was additionally diluted four times to be below ULOQ. About 1.5 − 2 µL of the sample was injected onto an ACQUITY UPLC BEH C18 1.7 µM (2.1 ×50 mm) (Waters Corporation, Taunton, Massachusetts, USA) column.

### LC–MS/MS analysis and data processing

The bioanalysis of samples from both in vivo and in vitro studies was performed using reversed-phase ultraperformance liquid chromatography (UPLC) (ACQUITY, Waters Corporation, Taunton, Massachusetts, USA) followed by tandem mass spectrometry (Xevo TQ-S micro, Waters, UK). Detection and quantitation of analytes were performed using multiple-reaction monitoring (MRM) to monitor parent → product ion (*m/z*) transitions (Supplementary Table 7) in positive electrospray (ES + ) ionization mode. The source-dependent parameters used for all compounds were: capillary voltage: 1.0 kV, source temperature: 150 °C, desolvation temperature: 600 °C, desolvation gas flow: 1000 L h^−1^, and cone gas flow: 10 L h^−1^. The mobile phases used to detect risperidone, clozapine, olanzapine, and the corresponding internal standards were MPA and 90% acetonitrile in 0.1% FA as elution solvent B (MPB), and were delivered at 0.3 mL min^−1^. Gradient elution was performed using the following steps: (i) a step gradient from 10% MPB to 50% MPB from 0 to 4.5 min, (ii) a step gradient from 50% MPB to 100% MPB from 4.5 to 4.7 min, and (iii) 100% MPB from 4.7 to 5.2 min, followed by (iv) reequilibration of the column with 10% MPB from 5.2 to 5.5 min. Sample processing and quantification were performed using MassLynx 4.2 and TargetLynx (Waters, UK), respectively. The lowest nonzero standard in the calibration curve was defined as the lower limit of quantification (LLOQ). Blanks, calibration curves and quality controls were included in all analytical runs. All standard curves weighted as 1/*x*^2^ were accepted at *R*^2^ values ≥0.99. Standard deviations for the responses of internal standards were below 6% in all analytical runs. Over 90% of quality controls were within ± 10% of the nominal values.

### Validation and cross-validation of qMSI-uD

The qMSI-uD method was validated in terms of linearity and range, coefficient of determination (*R*^2^), limit of detection (LOD), within-run and interday accuracy, and precision, selectivity, and specificity by applying the requirements specified in the American authorities’ guidelines for the validation of bioanalytical methods [36]. For data evaluation, a linear-regression model was used, tested using the *F*-test, and accepted at *Significance F* < 0.05. Limits of detection (LOD) were calculated using the LINEST function based on the expression *LOD* = *3.3x σ/S*, where *S* is the slope of the calibration curve and *σ* is the standard deviation of the *y*-intercept. Limits of quantitation (LOQ) were defined as the lower limits of quantitation (LLOQ) for tissues, which were taken to equal the concentration of the most dilute calibration standard giving a nonzero signal. The LLOQs for risperidone, clozapine, and olanzapine were 103, 115, and 113 ng g^−1^, respectively. For plasma, LLOQs of 50 (risperidone), and 10 ng mL^−1^ (clozapine and olanzapine) were accepted, while an LLOQ of 5 ng mL^−1^ was applied for all three drugs in plasma. The method’s selectivity was evaluated using blank and LLOQ samples spotted on tissue samples. Specificity was evaluated based on the resolving power of the FTICR instrument. Within-run and interday accuracy and precision were evaluated for tissue samples based on relative standard deviation (RSD) of low (103, 115, and 1144 ng g^−1^ for risperidone, clozapine, and olanzapine, respectively) and high (411, 1147, and 4579 ng g^−1^) level quality control (QC) samples, reflecting a relevant concentration range closer to the expected experimental total concentrations for each drug. Within-run accuracy and precision for plasma samples were evaluated based on the RSDs of low (50 ng mL^−1^) and high (200 ng mL^−1^) level QC samples. For buffer, the low levels for QC samples were chosen as 5 ng mL^−1^ (risperidone and clozapine) and 100 ng mL^−1^ (olanzapine), and the high levels were 100 ng mL^−1^ (risperidone and clozapine) and 1000 ng mL^−1^ (olanzapine). Stability was evaluated for 24 and 72 hours during and between the measurements based on the RSDs of all measured standard solutions.

### Pharmacokinetic basis for mapping the regional and subregional transport of an unbound drug across the BBB using MALDI-qMSI

The extent of drug transport across the BBB is characterized by the pharmacokinetic parameter K_p,uu,brain_ [11, 12], i.e., the ratio of the unbound drug’s concentration in the brain to that in the plasma. This parameter measures the net flux of the unbound drug across the BBB, and its magnitude indicates the direction and efficiency of the BBB transport processes. If K_p,uu,brain_ exceeds unity, the dominant transport process at the BBB is active uptake, whereas a value below unity indicates predominant efflux. K_p,uu,brain_ is computed using the following expression:

3 $${{{{{\mathrm{K}}}}}}_{{{{{\mathrm{p,uu,brain}}}}}} = \frac{{{{{{{\mathrm{AUC}}}}}}_{{{{{\mathrm{u,brain}}}}}}}}{{{{{{{\mathrm{AUC}}}}}}_{{{{{\mathrm{u,plasma}}}}}}}} = \frac{{{{{{{\mathrm{C}}}}}}_{{{{{\mathrm{u,brain,ss}}}}}}}}{{{{{{{\mathrm{C}}}}}}_{{{{{\mathrm{u,plasma,ss}}}}}}}}$$

Here, AUC_u,brain_ and AUC_u,plasma_ are the areas under the curve (AUC) of the unbound drug concentration—time profile for the brain and the plasma; alternatively, C_u,brain,ss_ and C_u,plasma,ss_ (the steady-state unbound drug concentrations in the brain and plasma) can be used.

The CMA is a method for evaluating the extent of BBB transport based on K_p,uu,brain_ that has proven to be effective in the evaluation and selection of novel CNS drug candidates [26]. Importantly, it can also be used to estimate the extent of BBB transport in specific regions of interest (ROI) within the brain [25]. The methodological platform of CMA–ROI includes (i) in vivo neuroPK studies performed in rodents followed by measurement of partitioning coefficients (K_p,ROI_) in discrete brain regions, (ii) in vitro drug tissue-binding studies for determination of the fraction of unbound drug in plasma (f_u,plasma_), and (iii) in vitro brain slice studies for evaluation of the unbound volume of distribution in discrete brain regions (V_u,ROI_). The extent of unbound drug transport across the BBB in the various CNS regions (K_p,uu,ROI_) is then determined from the experimentally determined K_p,ROI_, V_u,ROI_, and f_u,plasma_ using the following equation:

4 $${{{{{\mathrm{K}}}}}}_{{{{{\mathrm{p,uu,ROI}}}}}} = \frac{{{{{{{\mathrm{K}}}}}}_{{{{{\mathrm{p,ROI}}}}}}}}{{{{{{{\mathrm{V}}}}}}_{{{{{\mathrm{u,ROI}}}}}} \cdot {{{{{\mathrm{f}}}}}}_{{{{{\mathrm{u,plasma}}}}}}}}$$

where K_p,ROI_ is the ratio of the total CNS regional concentration of the drug to its total plasma concentration under steady-state conditions.

Unfortunately, K_p,uu,ROI_ cannot be estimated in small regions and subregions due to multiple methodological and technical problems, including the difficulty of dissecting small regions and performing bioanalysis with the resulting low sample volumes. By qMSI-uD approach, we overcome existing limitations. The underlying hypothesis of qMSI-uD is that the ratio of the total brain concentrations obtained from the in vivo neuroPK study and the in vitro brain-slice assay corresponds to the extent of BBB transport of the unbound drug (Fig. 1).

Rewriting the second part of Eq. 3 by representing C_u,brain,ss_ as the quotient of C_tot,brain,ss_ and V_u,brain_, we obtain:

5 $${{{{{\mathrm{K}}}}}}_{{{{{\mathrm{p,uu,brain}}}}}} = \frac{{{{{{{\mathrm{C}}}}}}_{{{{{\mathrm{tot,brain,ss}}}}}}}}{{{{{{{\mathrm{V}}}}}}_{{{{{\mathrm{u,brain}}}}}} \cdot {{{{{\mathrm{C}}}}}}_{{{{{\mathrm{u,plasma,ss}}}}}}}}$$

By incorporating Eq. 2, Eq. 5 can be rewritten as

6 $${{{{{\mathrm{K}}}}}}_{{{{{\mathrm{p,uu,brain}}}}}} = \frac{{{{{{{\mathrm{C}}}}}}_{{{{{\mathrm{tot,brain,ss}}}}}} \cdot {{{{{\mathrm{C}}}}}}_{{{{{\mathrm{u,buffer,ss}}}}}}}}{{{{{{{\mathrm{C}}}}}}_{{{{{\mathrm{tot,brain.slice,ss}}}}}} \cdot {{{{{\mathrm{C}}}}}}_{{{{{\mathrm{u,plasma,ss}}}}}}}}$$

When the unbound plasma and buffer concentration are equal, Eq. 6 reduces to:

7 $${{{{{\mathrm{K}}}}}}_{{{{{\mathrm{p,uu,brain}}}}}} = \frac{{{{{{{\mathrm{C}}}}}}_{{{{{\mathrm{tot,brain,ss}}}}}}}}{{{{{{{\mathrm{C}}}}}}_{{{{{\mathrm{tot,brain.slice,ss}}}}}}}}$$

When applied to a specific region of interest in the brain, Eq. 7 becomes

8 $${{{{{\mathrm{K}}}}}}_{{{{{\mathrm{p,uu,ROI}}}}}} = \frac{{{{{{{\mathrm{C}}}}}}_{{{{{\mathrm{tot,brainROI,ss}}}}}}}}{{{{{{{\mathrm{C}}}}}}_{{{{{\mathrm{tot,brain.sliceROI.ss}}}}}}}}$$

If the plasma and buffer concentrations of the drug differ, a correction factor (CF) must be computed and applied. If K_p,uu,ROI_ and V_u,brain_ are concentration-independent within the concentration range of interest, the CF can be taken to be the ratio of the unbound plasma and buffer concentrations, which can be determined in an independent experiment.

9 $${{{{{\mathrm{K}}}}}}_{{{{{\mathrm{p,uu,ROI}}}}}} = {{{{{\mathrm{CF}}}}}}\frac{{{{{{{\mathrm{C}}}}}}_{{{{{\mathrm{tot,brainROI,ss}}}}}}}}{{{{{{{\mathrm{C}}}}}}_{{{{{\mathrm{tot,brain.sliceROI.ss}}}}}}}}$$

The method relies on several implicit and explicit assumptions, which are presented below. These assumptions define the method’s boundaries and the validity of any inference drawn from the obtained results. One of the method’s implicit assumptions is that steady-state conditions are established in both experimental settings. Because any violation of this assumption will significantly affect the results obtained, the achievement of a steady state must be confirmed experimentally in both settings. Of the explicit assumptions arising from heuristic principles, the most critical ones are (i) low-molecular-weight drugs (ca. 500 Da) are transported in accordance with the free-drug hypothesis, i.e., only unbound and nonionized molecules can cross membranes, (ii) only unbound drug is present in the virtually protein-free brain interstitial fluid (ISF), (iii) overall CNS drug disposition is not affected by brain drug metabolism and brain bulk flow, and (iv) the BBB transport and intra-brain distribution of a drug is concentration-independent in the concentration range of interest, enabling the application of a correction factor according to Eq. 9 if needed. The proposed method is thus valid for small-molecular-weight drugs that satisfy these assumptions.

### Statistical analysis

All statistical analyses were performed using GraphPadPrism 6.04 for Windows (GraphPad Software, San Diego, California, USA). Descriptive statistics are reported in the form of means and standard deviations (mean ± SD). The Gaussian distribution of the data was tested using the Shapiro–Wilk normality test. If the normality test was passed, parametric statistical tests were used for further data analysis. Outliers in the data were identified by nonlinear regression using the ROUT method and excluded from further analysis. Differences between the mean values of C_tot,brain,ss_, C_tot,brain.slice,ss_, C_tot,brainROI,ss_, C_tot,br.sliceROI,ss_, K_p_, K_p,ROI_, K_p,uu,brain_, K_p,uu,ROI_ determined by qMSI-uD were assessed by ordinary one-way analysis of variance, after which Dunnett’s multiple-comparison test was used to evaluate differences between the neuroPK parameters determined at the total and region-annotated levels. Differences between the mean values of C_tot,plasma,ss_, C_tot,buffer,ss_, C_tot,brain,ss_, C_tot,brain.slice,ss_, K_p,brain_, K_p,uu,brain_ determined by LC–MS/MS and MALDI-qMSI were assessed by the unpaired two-tailed *t*-test. Statistical analyses were not performed for groups with fewer than three samples.

## Supplementary information

Supplementary Tables and Figures
