# Supplementary material for: Neuropharmacokinetic visualization of regional and subregional unbound antipsychotic drug transport across the blood–brain barrier
Source: Mol Psychiatry. 2021 Sep 3;26(12):7732–45. doi: 10.1038/s41380-021-01267-y (PMC8872980; doi:10.1038/s41380-021-01267-y)
Supplement: Supplementary file 1 — Supplementary Tables and Figures [file 41380_2021_1267_MOESM1_ESM.pdf]

Neuropharmacokinetic visualization of regional and subregional unbound antipsychotic drug transport across the blood-brain barrier

Dominika Luptáková[1], Ph.D., Theodosia Vallianatou[1], Ph.D., Anna Nilsson[1,2], Ph.D., Reza Shariatgorji[1,2], Ph.D., Margareta Hammarlund-Udenaes[3], Ph.D., Irena Loryan[3]*, Ph.D., Per E. Andrén[1,2]*, Ph.D.

[1] Department of Pharmaceutical Biosciences, Medical Mass Spectrometry Imaging, Uppsala University, BMC 591, SE-75124 Uppsala, Sweden

[2] Science for Life Laboratory, Spatial Mass Spectrometry, Uppsala University, BMC 591, SE-75124 Uppsala, Sweden

[3] Department of Pharmacy, Translational PKPD Research Group, Uppsala University, BMC 580, SE-75123 Uppsala, Sweden; Associate member of SciLifeLab.

* Corresponding authors:
irena.loryan@farmaci.uu.se and per.andren@farmbio.uu.se

Supplementary Table 1. Validation of the MALDI-qMSI method for bioanalysis of total drug concentrations in rat brain tissues obtained from *in vivo* neuroPK and *in vitro* brain slice experiments.

Analytical characteristics used to validate the method include the linearity and range, coefficient of determination (R^2), limit of detection (LOD), accuracy and precision (expressed as relative standard deviation, RSD). Characteristics are based on measurements of the calibration standards at five (for risperidone) or six (for clozapine and olanzapine) levels, quality controls (QC), and blanks performed at the beginning, in the middle, and at the end of each MALDI-qMSI acquisition on Day 1, 2, and 3. Accuracy and precision parameters were determined using low- and high-level QC samples. Validation parameters were evaluated separately for risperidone (**a**, **b**), clozapine (**c**, **d**), and olanzapine (**e**, **f**) for both experimental settings.

a

Risperidone		NeuroPK study in rats			Rat brain slice study		
		Day 1	Day 2	Day 3	Day 1	Day 2	Day 3
**Linear regression equation**	**a**	6.5526	7.1588	7.1751	7.2082	6.7147	6.8799
**(y = ax + b)**	**b**	- 0.0357	- 0.0158	- 0.0582	- 0.0051	0.001	-0.0179
**Range (ng g^{-1})**		103 – 2056					
**Coefficient of determination (R^2)**		0.9996	0.9969	0.9988	0.9997	0.9992	0.9998
**LOD (ng g^{-1})**		23.2			31.2		

b

Risperidone	QC level (ng g^{-1})	NeuroPK study in rats		Rat brain slice study	
		Accuracy (%)	Precision (RSD, %)	Accuracy (%)	Precision (RSD, %)
**Day 1**	103	110	8	91	4
	411	87	6	79	4
**Day 2**	103	97	16	97	9
	411	88	24	80	6
**Day 3**	103	115	5	104	8
	411	88	13	80	6
**Inter-day (mean)**	103	107	10	97	7
	411	88	15	79	5

c

Clozapine		NeuroPK study in rats			Rat brain slice study		
		Day 1	Day 2	Day 3	Day 1	Day 2	Day 3
Linear regression equation	a	6.3033	6.1878	5.9083	6.8289	8.0282	6.8314
(y = ax + b)	b	- 0.1253	- 0.0834	- 0.1096	- 0.1008	- 0.1238	- 0.0439
Range (ng g^{-1})		115 – 4588					
Coefficient of determination (R^2)		0.9989	0.9996	0.9993	0.9997	0.9998	0.9996
LOD (ng g^{-1})		87.0			55.2		

d

Clozapine	QC level (ng g^{-1})	NeuroPK study in rats		Rat brain slice study	
		Accuracy (%)	Precision (RSD, %)	Accuracy (%)	Precision (RSD, %)
Day 1	115	100	5	112	8
	1147	94	2	89	4
Day 2	115	128	8	102	9
	1147	100	3	78	8
Day 3	115	122	2	90	8
	1147	87	6	88	9
Inter-day (mean)	115	116	5	101	8
	1147	94	4	85	7

e

Olanzapine		NeuroPK study in rats			Rat brain slice study		
		Day 1	Day 2	Day 3	Day 1	Day 2	Day 3
Linear regression equation	a	2.4803	2.865	2.0406	2.394	2.6131	2.5316
(y = ax + b)	b	- 0.0099	- 0.0321	0.05	- 0.0106	0.0182	0.0486
Range (ng g^{-1})		113 – 4550					
Coefficient of determination (R^2)		0.9999	0.9997	0.9962	0.9994	0.9989	0.9927
LOD (ng g^{-1})		34.8			47.8		

f

Olanzapine	QC level (ng g^{-1})	NeuroPK study in rats		Rat brain slice study	
		Accuracy (%)	Precision (RSD, %)	Accuracy (%)	Precision (RSD, %)
**Day 1**	1144	87	4	92	7
	4579	90	8	88	2
**Day 2**	1144	92	9	94	9
	4579	81	9	89	9
**Day 3**	1144	88	12	94	16
	4579	108	17	89	8
**Inter-day (mean)**	1144	96	8	93	11
	4579	94	11	89	6

Supplementary Table 2. Validation of the MALDI-qMSI method used for bioanalysis of total drug concentrations in rat plasma and buffer samples.

The plasma and buffer samples were spotted on a ground steel MALDI plate and the MALDI-qMSI method was separately validated for these matrices based on the following analytical characteristics: **a**, linearity and range, coefficient of determination (R^2), and limit of detection (LOD), as well as **b**, accuracy and precision. Characteristics are based on measurements of standards at six (for plasma) or eight (buffer) levels, quality controls (QCs), and blanks performed at the beginning, in the middle, and at the end of each MALDI-qMSI acquisition. Accuracy and precision parameters were determined using low- and high-QC level samples. All values represent an average of three independent measurements within a day.

a

		Plasma (NeuroPK study in rats)			Buffer (Rat brain slice study)		
		**Risperidone**	**Clozapine**	**Olanzapine**	**Risperidone**	**Clozapine**	**Olanzapine**
**Linear regression equation (y = ax + b)**	**a**	6827.9	3247.7	3618.6	22245	4942.2	4097.1
	**b**	11.854	- 48.664	10.8	26.644	11.933	- 31.536
**Range (ng mL^{-1})**		50 - 1000	10 - 1000	10 - 1000	5 - 100	5 - 100	5 - 1000
**Coefficient of determination (R^2)**		0.9994	0.9997	0.9987	0.9995	0.9987	0.9972
**LOD (ng mL^{-1})**		18.4	3.4	4.1	2.1	2.8	4.7

b

	Plasma (NeuroPK study in rats)			Buffer (Rat brain slice study)		
	**QC (ng mL^{-1})**	**Accuracy (%)**	**Precision (RSD %)**	**QC (ng mL^{-1})**	**Accuracy (%)**	**Precision (RSD %)**
**Risperidone**	50	120.7	4.5	5	124.9	5.1
	200	92.5	2.1	100	95.3	2.3
**Clozapine**	50	101.4	2.6	5	105.7	0.4
	200	92.3	0.5	100	90.1	0.8
**Olanzapine**	50	88.6	2.6	100	86.3	1.1
	200	93.3	0.2	1000	105.2	0.4

Supplementary Table 3. Whole brain and region-annotated total concentrations of risperidone, clozapine, and olanzapine determined by MALDI-qMSI in brain tissue samples from *in vivo* neuroPK and *in vitro* brain slice experiments.

Total drug concentrations were determined in coronal sections of brain tissues (distance from bregma ca 2.28 mm) from *in vivo* and *in vitro* experiments conducted under steady-state conditions. The total concentration of a drug in the entire coronal section is referred to as the whole brain concentration. All tissues were measured three times over three days. Results are reported as means ± standard deviation of the mean together with the corresponding numbers of biological/technical replicates. Abbreviations: cc, corpus callosum; ec, external capsule; aca, anterior commissure; CgCx, cingulate cortex; MCx, motor cortex; SCx, somatosensory cortex; InCx, insular cortex; Pir, piriform cortex; CPu, caudate putamen; NAc, nucleus accumbens; LS, lateral septum. * The NAc region was identified in only one out of three brain slice samples.

**Concentration at steady state (ng g^{-1})**	**Risperidone**		**Clozapine**		**Olanzapine**	
	**NeuroPK study in rats**	**Rat brain slice study**	**NeuroPK study in rats**	**Rat brain slice study**	**NeuroPK study in rats**	**Rat brain slice study**
**Whole brain**	233.4±36.8 (5/15)	681.2±31.1 (3/9)	621.7±67.6 (6/18)	1158±148 (3/9)	724.8±164.3 (6/18)	1418±208 (3/9)
**cc**	96.2±17.8 (5/15)	360.5±22.9 (3/9)	351.8±41.1 (6/18)	597.4±71.9 (3/9)	208.8±45.6 (6/18)	544.7±89.7 (2/6)
**ec**	115.1±16.7 (5/15)	451.2±51.3 (3/9)	407.1±51 (6/18)	767.5±146.8 (3/9)	337.9±76.8 (6/18)	662±96 (3/9)
**aca**	165.6±25.1 (5/15)	432.3±73.8 (2/6)	440.4±68.8 (6/18)	698.5±69.6 (2/6)	314.7±49.7 (4/12)	461±106 (2/6)
**CgCx**	254±55 (5/15)	784.6±85.8 (3/9)	738.9±86.2 (6/18)	1349±121 (3/9)	877.1±179.2 (6/18)	1881±237 (3/9)
**MCx**	222.6±42.5 (5/15)	829.9±64.8 (3/9)	677.8±85.2 (6/18)	1289±143 (3/9)	880±193 (6/18)	1745±465 (3/9)
**SCx**	218.9±35.8 (5/15)	698.8±78.5 (3/9)	660.7±79.3 (6/18)	1266±121 (3/9)	781.9±184.9 (6/18)	1375±266 (3/9)
**InCx**	301.9±59.5 (5/15)	937.3±111.9 (3/9)	772.9±93.2 (6/18)	1541±173 (3/9)	1063±239 (6/18)	2374±427 (3/9)
**Pir**	255±52 (5/15)	610.6±148.3 (3/7)	633.7±63.6 (6/18)	1521±243 (3/8)	762.8±198.5 (6/18)	1383±253 (3/9)
**CPu**	267.5±47.3 (5/15)	760.6±76.2 (3/9)	660.1±77.9 (6/18)	1166±219 (3/9)	740.8±185.9 (6/18)	1375±292 (3/9)
**NAc**	274.9±46.5 (5/15)	795.5±90.5 (2/6)	648.9±87.3 (6/18)	1397±202 (2/6)	884.9±168.9 (4/12)	1871±96 (1/3)*
**LS**	233.3±34.3 (5/15)	636.9±132.1 (2/6)	587.9±92.5 (4/12)	1416±114 (3/9)	681.9±180.2 (6/18)	2517±638 (2/6)

Supplementary Table 4. Results of Dunnett's multiple comparison tests comparing the mean whole brain and region-annotated total concentrations of risperidone, clozapine and olanzapine in brain tissue sections from *in vivo* neuroPK and the *in vitro* brain slice experiments.

Total concentration of a drug in the entire coronal section is referred as whole brain concentration, and it was used as a reference group in multiple comparison. Results are expressed as multiplicity adjusted *P* values resulting from Dunnett's multiple comparison test (GraphPad Prism version 7.05 for Windows, GraphPad Software, San Diego, California USA, www.graphpad.com). The results were considered as significant if P value ≤ 0.05. Abbreviations: cc, corpus callosum; ec, external capsule; aca, anterior commissure; CgCx, cingulate cortex; MCx, motor cortex; SCx, somatosensory cortex; InCx, insular cortex; Pir, piriform cortex; CPu, caudate putamen; NAc, nucleus accumbens; LS, lateral septum; NA, not analyzed due to the number of samples lower than three in a group.

	NeuroPK study in rats			Rat brain slice study		
	**Risperidone**	**Clozapine**	**Olanzapine**	**Risperidone**	**Clozapine**	**Olanzapine**
**cc**	0.0008	<0.0001	0.0008	0.0076	0.0565	NA
**ec**	0.002	0.0002	0.001	0.0558	0.0354	0.0201
**aca**	0.0406	0.0009	0.0014	NA	NA	NA
**CgCx**	0.3327	0.0003	0.1284	0.2895	0.4467	0.0014
**MCx**	0.0695	0.0079	0.0197	0.126	0.0694	0.6682
**SCx**	0.019	0.0157	0.1528	0.99	0.4584	0.9895
**InCx**	0.0054	0.0004	0.0014	0.1378	0.0652	0.0478
**Pir**	0.0714	0.6483	0.8929	0.96	0.2387	0.9999
**CPu**	0.0039	0.0549	0.8836	0.2537	0.9998	0.9906
**NAc**	0.007	0.5754	0.2088	NA	NA	NA
**LS**	>0.9999	0.1001	0.9929	NA	0.9555	NA

Supplementary Table 5. Summary on the extent of unbound risperidone, clozapine, and olanzapine transport across the whole brain and regional blood-brain barrier determined by qMSI-uD.

$K_{p,uu}$ values were calculated as the ratio of the total steady-state concentrations of a drug *in vivo* (either whole brain or region annotated total concentration) to the whole brain total steady-state concentration of the same drug observed in an *in vitro* brain slice study, multiplied by a correction factor (Eq. 9, Methods). The total concentration of a drug in the entire coronal section is referred as the whole brain concentration. $K_{p,uu}$ results are expressed as means ± standard deviation. Abbreviations: n, total number of biological replicates in the *in vivo* neuroPK study; cc, corpus callosum; ec, external capsule; aca, anterior commissure; CgCx, cingulate cortex; MCx, motor cortex; SCx, somatosensory cortex; InCx, insular cortex; Pir, piriform cortex; CPu, caudate putamen; NAc, nucleus accumbens; LS, lateral septum. *total number of biological replicates with identified LS brain region (n=4 for clozapine), and aca and NAc brain regions (n=4 for olanzapine)

$K_{p,uu}$	Risperidone, n=5	Clozapine, n=6	Olanzapine, n=6
**Whole brain**	0.10±0.02	0.31±0.03	0.82±0.19
**cc**	0.08±0.01	0.34±0.04	0.62±0.13
**ec**	0.07±0.01	0.31±0.04	0.82±0.19
**aca**	0.11±0.02	0.36±0.05	1.10±0.15*
**CgCx**	0.09±0.02	0.32±0.04	0.75±0.15
**MCx**	0.08±0.01	0.30±0.04	0.81±0.18
**SCx**	0.09±0.01	0.30±0.04	0.91±0.22
**InCx**	0.09±0.02	0.29±0.03	0.72±0.16
**Pir**	0.12±0.02	0.24±0.02	0.88±0.23
**CPu**	0.10±0.02	0.33±0.04	0.87±0.22
**NAc**	0.10±0.02	0.27±0.03	0.76±0.13*
**LS**	0.11±0.01	0.27±0.04*	0.44±0.16

Supplementary Table 6. Results of multiple comparison tests comparing whole brain and region-annotated $K_{p,uu}$ values (Dunnett's multiple comparison test) or different region-annotated $K_{p,uu}$ values (Tukey multiple comparison test) for risperidone, clozapine and olanzapine.

Comparisons of mean whole brain and mean region-annotated values for the extent of unbound **a,** risperidone, **b,** clozapine and **c,** olanzapine transport across the blood-brain barrier (BBB) were performed using the Dunnett's multiple comparison test. Comparisons of the mean extent of unbound drug BBB transport between regions and subregions were performed with the Tukey multiple comparison test. Results are expressed as multiplicity-adjusted *P* values resulting from Dunnett's and Tukey multiple comparison tests (GraphPad Prism version 7.05 for Windows, GraphPad Software, San Diego, California USA, www.graphpad.com). The results were considered significant if $P \leq 0.05$. Abbreviations: cc, corpus callosum; ec, external capsule; aca, anterior commissure; CgCx, cingulate cortex; MCx, motor cortex; SCx, somatosensory cortex; InCx, insular cortex; Pir, piriform cortex; CPu, caudate putamen; NAc, nucleus accumbens; LS, lateral septum. $^{+}$, The Tukey multiple comparison test for olanzapine was performed without considering the aca and NAc brain regions because the numbers of $K_{p,uu,ROI}$ values for these regions differed from those for other regions. $^{++}$, Tukey multiple comparison tests including aca and NAc brain regions were performed without considering rats for which aca and NAc regions were not visible in the tissue section. *, The Tukey multiple comparison test for clozapine was performed without considering the LS brain region because the number of $K_{p,uu,ROI}$ values for this region differed from those for other regions. **, Tukey multiple comparison tests including the LS brain region were performed without considering rats, for which LS was not visible in the tissue section.

a

Risperidone $K_{p,uu}$	cc	ec	CgCx	MCx	SCx	InCx	Pir	CPu	aca	NAc	LS
**Whole brain**	0.0013	0.0088	0.4509	0.0004	0.0075	0.0339	0.0175	0.2232	0.537	0.9974	0.022
**cc**		0.8606	0.1168	0.9998	0.0073	0.0292	0.01	0.0074	0.0604	0.006	0.0007
**ec**	0.8606		0.1698	0.9132	0.0577	0.0815	0.0254	0.0179	0.0192	0.017	0.004
**CgCx**	0.1168	0.1698		0.0659	0.9798	>0.9999	0.01	0.4301	0.6564	0.4256	0.107
**MCx**	0.9998	0.9132	0.0659		0.002	0.0217	0.0058	0.0046	0.0792	0.0031	0.0003
**SCx**	0.0073	0.0577	0.9798	0.002		0.9155	0.0197	0.037	0.3638	0.1388	0.0004
**InCx**	0.0292	0.0815	>0.9999	0.0217	0.9155		0.0147	0.0248	0.417	0.4359	0.0465
**Pir**	0.01	0.0254	0.01	0.0058	0.0197	0.0147		0.0658	0.9964	0.0724	0.2954
**CPu**	0.0074	0.0179	0.4301	0.0046	0.037	0.0248	0.0658		0.8977	0.9945	0.4299
**aca**	0.0604	0.0192	0.6564	0.0792	0.3638	0.417	0.9964	0.8977		0.7709	0.998
**NAc**	0.006	0.017	0.4256	0.0031	0.1388	0.4359	0.0724	0.9945	0.7709		0.3103
**LS**	0.0007	0.004	0.107	0.0003	0.0004	0.0465	0.2954	0.4299	0.998	0.3103	

b

Clozapine $K_{p,uu}$	cc*	ec*	aca*	CgCx*	MCx*	SCx*	InCx*	Pir*	Cpu*	Nac*	LS**
**Whole brain**	0.0163	0.9978	0.052	0.3848	0.3429	0.104	0.0155	0.0003	0.0888	0.0118	0.0076
**cc***		0.0379	0.6839	0.1537	0.0086	0.0163	0.0115	0.0003	0.7011	0.0043	0.0338
**ec***	0.0379		0.026	0.9631	>0.9999	0.9997	0.7256	0.0032	0.5465	0.2828	0.0047
**aca***	0.6839	0.026		0.1257	0.0556	0.0431	0.031	0.0036	0.2805	0.0328	0.0076
**CgCx***	0.1537	0.9631	0.1257		0.025	0.0008	0.0003	0.0014	0.0503	0.0358	0.037
**MCx***	0.0086	>0.9999	0.0556	0.025		0.9219	0.0932	0.0039	0.0084	0.0923	0.1341
**SCx***	0.0163	0.9997	0.0431	0.0008	0.9219		0.0965	0.0037	0.0053	0.1085	0.0831
**InCx***	0.0115	0.7256	0.031	0.0003	0.0932	0.0965		0.0082	<0.0001	0.4981	0.3862
**Pir***	0.0003	0.0032	0.0036	0.0014	0.0039	0.0037	0.0082		0.0017	0.0926	0.2044
**Cpu***	0.7011	0.5465	0.2805	0.0503	0.0084	0.0053	<0.0001	0.0017		0.0309	0.0588
**Nac***	0.0043	0.2828	0.0328	0.0358	0.0923	0.1085	0.4981	0.0926	0.0309		>0.9999
**LS****	0.0338	0.0047	0.0076	0.037	0.1341	0.0831	0.3862	0.2044	0.0588	>0.9999	

c

Olanzapine $K_{p,uu}$	cc^{+}	ec^{+}	aca^{++}	CgCx^{+}	MCx^{+}	SCx^{+}	InCx^{+}	Pir^{+}	Cpu^{+}	Nac^{++}	LS^{+}
**Whole brain**	0.0064	>0.9999	0.0218	0.6767	0.9995	0.0591	0.0339	0.6834	0.3136	0.2063	0.006
**cc^{+}**		0.0099	0.02	0.2553	0.0062	0.0088	0.0661	0.0207	0.0426	0.2821	0.0487
**ec^{+}**	0.0099		0.0708	0.9633	>0.9999	0.4024	0.1233	0.7034	0.9547	0.1986	0.0054
**aca^{++}**	0.02	0.0708		0.2993	0.1384	0.2804	0.0143	0.0733	0.1471	0.0462	0.0441
**CgCx^{+}**	0.2553	0.9633	0.2993		0.7559	0.3239	0.9994	0.746	0.6374	>0.9999	0.0337
**MCx^{+}**	0.0062	>0.9999	0.1384	0.7559		0.1456	0.2811	0.7787	0.9121	0.9909	0.0077
**SCx^{+}**	0.0088	0.4024	0.2804	0.3239	0.1456		0.0152	0.9873	0.8325	0.1998	0.0111
**InCx^{+}**	0.0661	0.1233	0.0143	0.9994	0.2811	0.0152		0.0465	0.1603	0.9626	0.0485
**Pir^{+}**	0.0207	0.7034	0.0733	0.746	0.7787	0.9873	0.0465		>0.9999	0.4777	0.0173
**Cpu^{+}**	0.0426	0.9547	0.1471	0.6374	0.9121	0.8325	0.1603	>0.9999		0.0463	0.0186
**Nac^{++}**	0.2821	0.1986	0.0462	>0.9999	0.9909	0.1998	0.9626	0.4777	0.0463		0.1824
**LS^{+}**	0.0487	0.0054	0.0441	0.0337	0.0077	0.0111	0.0485	0.0173	0.0186	0.1824	

Supplementary Table 7. Settings used for MS/MS analyses of risperidone, clozapine, and olanzapine and their deuterated analogs. The analysis was carried out in positive ionization mode, with a source temperature of 150 °C and a desolvation temperature of 600 °C.

Drug	Precursor-product ion pair (*m/z*)	Cone (V)	Collision energy (eV)
**Risperidone**	411.2→191.1	10	28
**Risperidone- d_4**	415.2→195.1	10	28
**Clozapine**	327.1 →270.1	10	22
**Clozapine- d_4**	331.1 →272.1	10	22
**Olanzapine**	313.1 → 256.1	10	22
**Olanzapine- d_8**	321.2 → 261.1	10	24

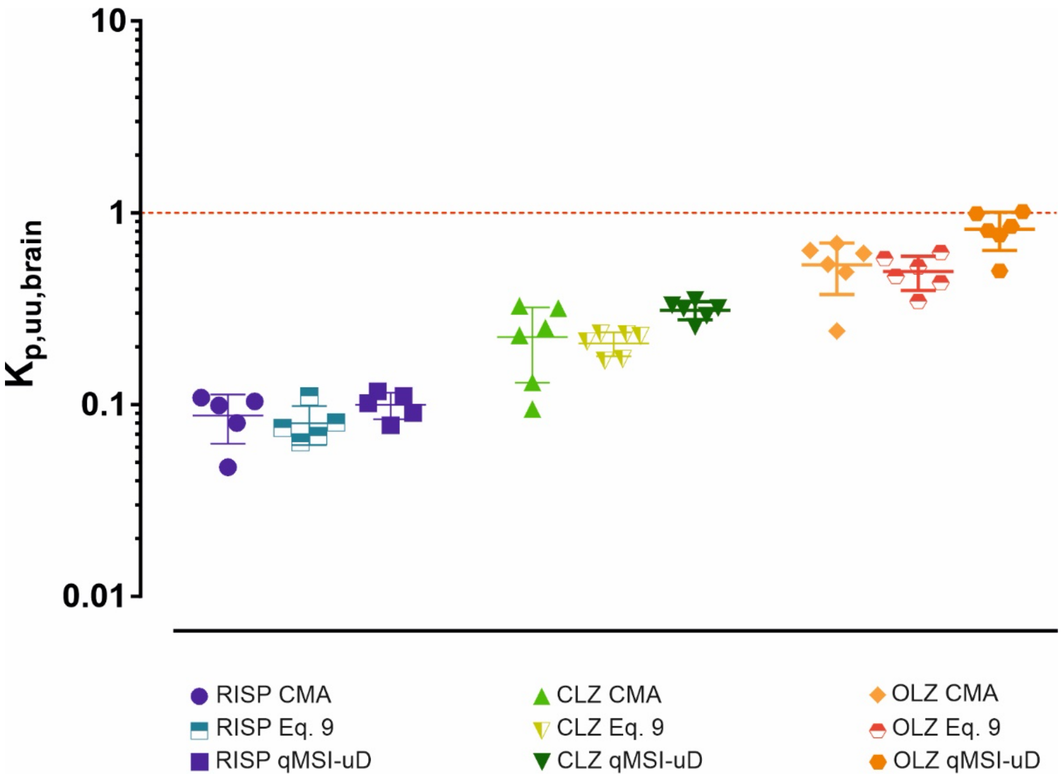

Supplementary Fig. 1. Performance of qMSI-uD compared to the conventional combinatory mapping approach (CMA) for determination of the extent of unbound drug BBB transport at the total brain level, $K_{p,uu,brain}$.

The qMSI-uD method was validated by comparison to conventional CMA. $K_{p,uu,brain}$ was assessed first using the established CMA (Methods, Eq. 5), then using the hypothesis represented by Eq. 9 (see Methods), and finally by the qMSI-uD method. Bioanalysis of the model drugs risperidone, clozapine, and olanzapine was performed in the whole brain (meaning a complete brain coronal section) either by the traditional LC-MS/MS method (in the first and second scenarios) or by MALDI-qMSI (third scenario). Results are shown in form of scatter dot plots. A $K_{p,uu,brain}$ value of unity is indicated by a red dashed line (the graph is plotted using a semilogarithmic scale). Error bars represent standard deviations from the mean. Abbreviations: RISP, risperidone; CLZ, clozapine; OLZ, olanzapine.

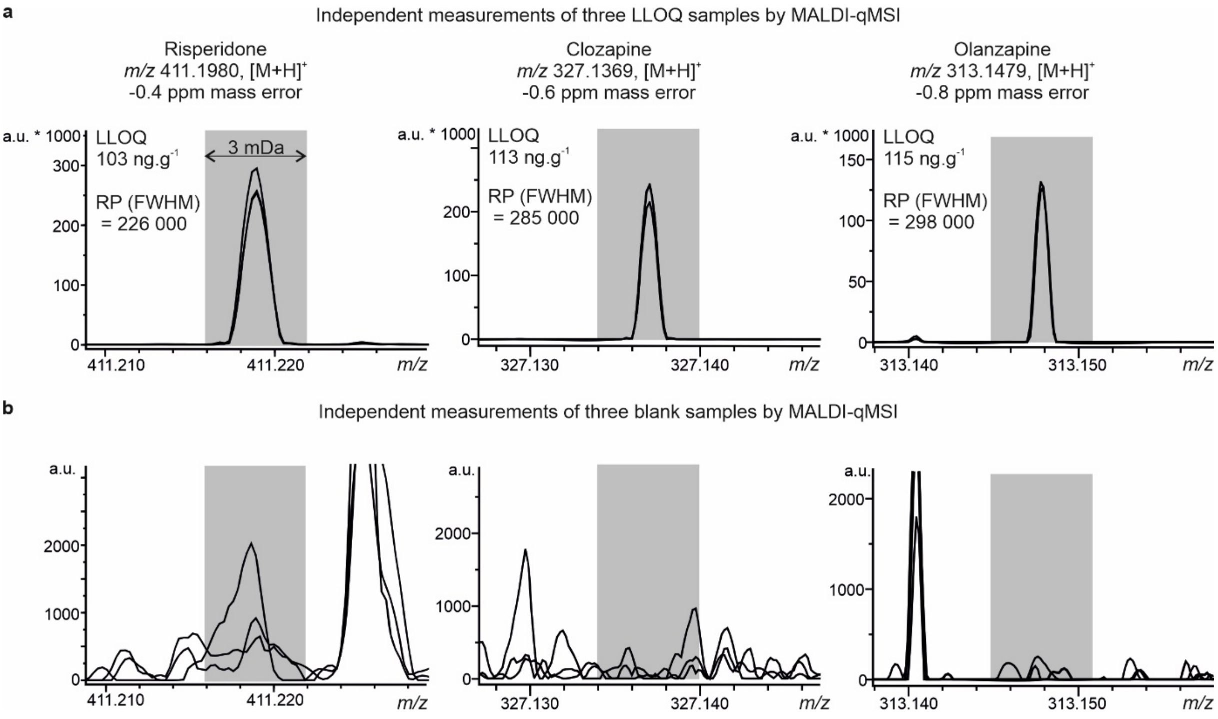

Supplementary Fig. 2. Specificity and selectivity of MALDI-qMSI for determination of the presence of risperidone, clozapine, and olanzapine in brain tissue.

The specificity and selectivity of the MALDI-qMSI method were determined individually for risperidone (left), clozapine (middle), and olanzapine (right). Its specificity was evaluated based on the non-zero calibration standard as **a,** the lowest limit of detection (LLOQ) and **b,** independent triplicate analyses of blank samples. No signals due to drugs were detected in blank samples. The intensity of the drug signals in the LLOQ samples was 100 times the noise level. The measured mass error of the MALDI-qMSI method was below 1 ppm for all drugs, demonstrating its selectivity. All data were detected with an instrument resolving power of up to 300 000. No interference peaks were detected in a narrow mass window of 3 ppm (gray area).

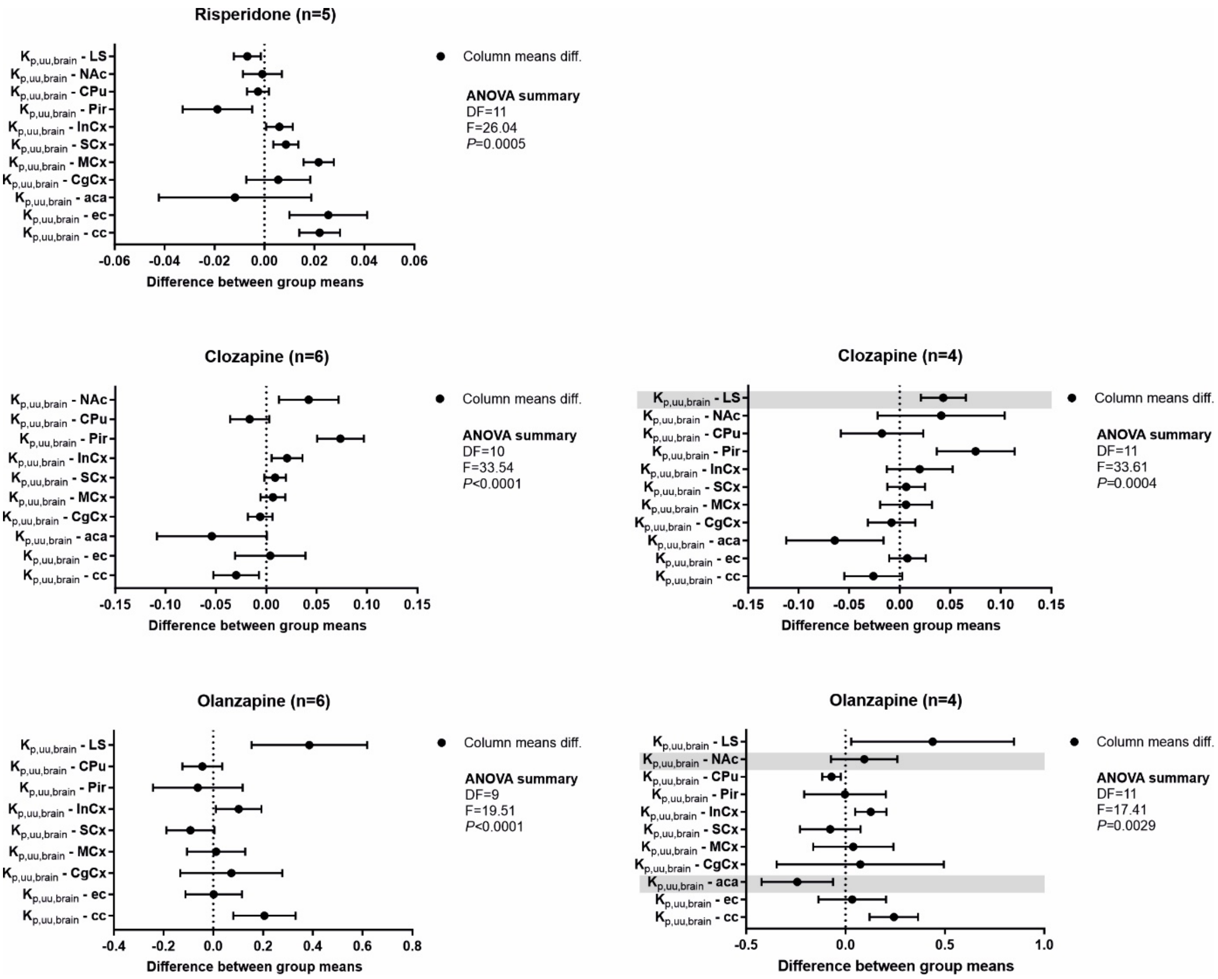

Supplementary Fig. 3. Dunnett's 95% confidence intervals for the differences between the mean whole brain $K_{p,uu,brain}$ and region-annotated $K_{p,uu,ROI}$ values of risperidone, clozapine and olanzapine.

One-way analysis of variance was performed to compare the extent of unbound risperidone (top), clozapine (middle), and olanzapine (bottom) transport across the blood-brain barrier in the whole brain (i.e. the entire brain section, $K_{p,uu,brain}$) to that in specific brain regions. Because the numbers of biological replicates in which certain brain regions could be identified (n) differed for both clozapine and olanzapine, two different statistical analyses were used. Analysis of variance was used for regions identified in all biological replicates (left) and in cases where all regions of interest were identified in at least some biological replicates (right). For the second analysis, only missing results from the first analysis were considered (gray shaded area). The results are presented in the form of 95% confidence intervals for the difference in the means for the groups being compared (a difference of 0 indicates no difference between groups). Additionally, the column means differences (indicated by filled black circles) are shown, representing the mean difference within a group of biological replicates. Abbreviations: cc, corpus callosum; ec, external capsule; aca, anterior commissure; CgCx, cingulate

cortex; MCx, motor cortex; SCx, somatosensory cortex; InCx, insular cortex; Pir, piriform cortex; CPu, caudate putamen; NAc, nucleus accumbens; LS, lateral septum; DF, degree of freedom; F, F-ratio; *P*, multiplicity-adjusted *P* values**.**

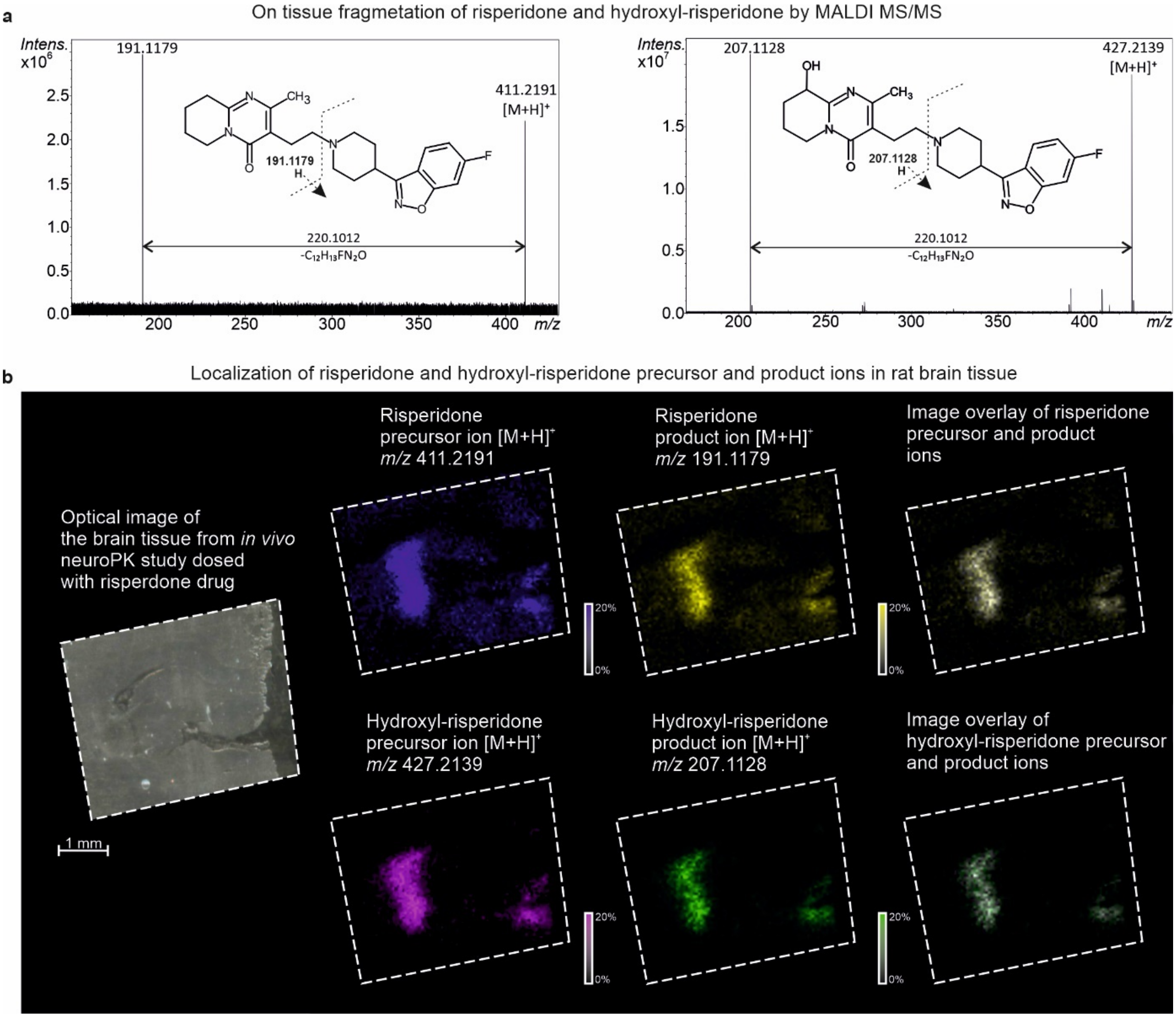

Supplementary Fig. 4. Localization of risperidone and hydroxyl-risperidone in rat brain visualized by MALDI MS/MS imaging.

A representative brain tissue sample from an animal dosed with risperidone during an *in vivo* neuroPK study was cryo-sectioned (coronal tissue section at bregma level -0.34 mm) was prepared as described in the Methods section (MALDI-qMSI sample preparation) and analyzed using MALDI MS/MS imaging. Data were acquired in the mass range 150 – 600 *m/z*. The MALDI MS/MS method was adjusted specifically for risperidone and hydroxyl-risperidone precursor ions. Precursor ions were selected with a mass window of 20 Da and fragmented by collision induced dissociation with a collision energy of 15 V. MS/MS imaging data were acquired with a 100 µm raster step. Acquired data were normalized against the product ion of the deuterated analogue and visualized using flexImaging software (Bruker Daltonics, v.5.0), and MS/MS spectra were processed using DataAnalysis software (Bruker Daltonics, v.5.0). **a**, Precursor ions of risperidone (*m/z* 411.2191, left) and hydroxyl-risperidone (*m/z* 427.2139, right) were fragmented. The main product ions were detected and identified at *m/z* 191.1179, and *m/z*

207.1128, corresponding to risperidone and hydroxyl-risperidone, respectively. **b**, MS/MS imaging revealed that risperidone and its metabolite hydroxyl-risperidone were mainly distributed in the lateral and third ventricles in the brain tissue section. The distribution of the product ions correlated well with those of precursor ions of the investigated compounds.

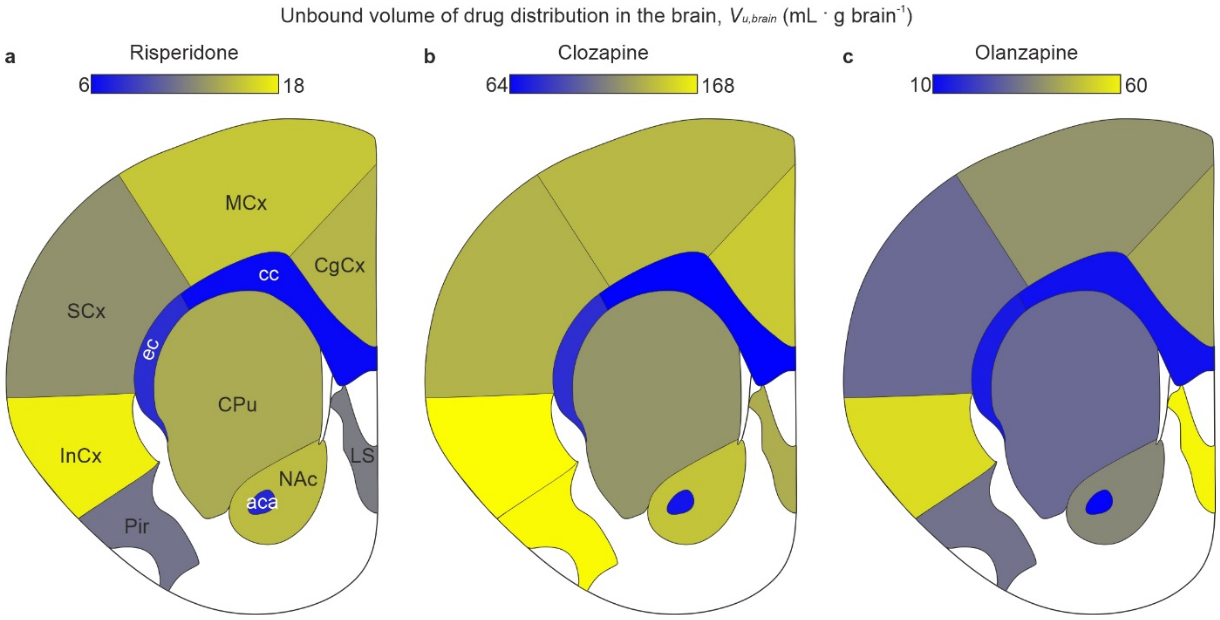

Supplementary Fig. 5. Visualizing of the unbound volume of distribution of risperidone, clozapine and olanzapine in the brain tissue.

The unbound volume of drug distribution ($V_{u,brain}$) was determined (see Eq. 2, Methods) after evaluation of the total drug concentration in brain slice and buffer under steady-state conditions using MALDI-qMSI in eleven selected brain regions and subregions for each drug. Individual region-annotated $V_{u,brain}$ values were used to create heat maps for **a**, risperidone, **b**, clozapine, and **c**, olanzapine. Abbreviations: cc, corpus callosum; ec, external capsule; aca, anterior commissure; CgCx, cingulate cortex; MCx, motor cortex; SCx, somatosensory cortex; InCx, insular cortex; Pir, piriform cortex; CPu, caudate putamen; NAc, nucleus accumbens; LS, lateral septum.

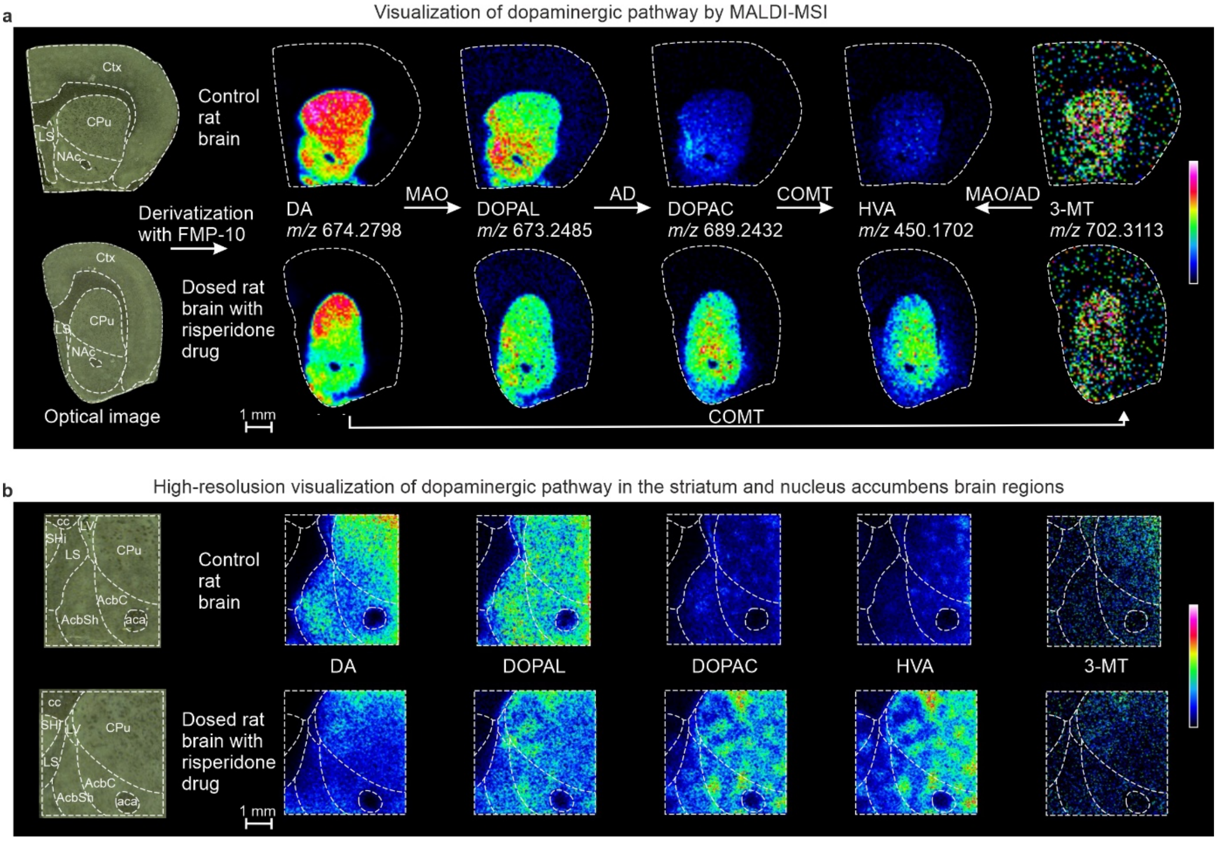

Supplementary Fig. 6. Visualization of dopaminergic pathway activity in the rat brain.

A representative control (n = 1) and risperidone-dosed (n =5) brain tissue samples from *in vivo* neuroPK study were cryo-sectioned at coronal bregma level 2.28 mm as described in the Method section (MALDI-qMSI sample preparation). Neurotransmitters were visualized upon derivatization with FMP-10 agent [1]. Briefly, after vacuum desiccation of the tissue sections for 40 minutes, FMP-10 solution (4.4 mM in 70% acetonitrile) was sprayed with an automatic TM-sprayer (HTX-Technologies 470 LLC, Chapel Hill, NC, USA) with the following conditions: the nozzle temperature of the spray head was set at 90 °C and the derivatization reagent was sprayed pneumatically (6 psi N_2) with a flow rate of 80 µL min^{-1} and a linear velocity of 110 cm min^{-1} in twenty passes with horizontal deposition and 2 mm track spacing. Then, samples were analyzed by MALDI MSI using a MALDI Fourier transform ion cyclotron resonance Solarix 7T 2Ω mass spectrometer (Bruker Daltonics, Bremen, Germany) equipped with a Smartbeam II 2 kHz laser and operated in positive ion mode. Data were acquired in the mass range 150 – 1000 *m/z* with adjusted operating conditions to reach the optimal ion intensity, and with 100 and 20 µm raster step for low and high-resolution experiments, respectively. Acquired data were normalized to root mean square and visualized using flexImaging software (Bruker Daltonics, v.5.0). **a**, Visualization of biochemical pathway of dopamine metabolism in the vehicle control (10% hydroxypropyl beta-cyclodextrin in saline) and risperidone-dosed rat brain tissues. Two different biochemical pathway of dopamine metabolism were visualized, either via activation of monoamine oxidase (MAO) or catechol-O-methyltransferase (COMT). Risperidone, as a dopamine receptor (preferentially D_2) antagonist, might

show effect on possible reuptake and metabolic degradation of dopamine causing increase abundance of metabolites in comparison with the control tissue. Remarkably, the results are in line with the findings from in vivo brain microdialysis study [2]. **b**, High-resolution images showing distribution of dopamine metabolites in striatum and nucleus accumbens brain regions and subregions. Dopamine (DA), 3,4-dihydroxyphenylacetaldehyde (DOPAL), 3,4-dihydroxyphenylacetic acid (DOPAC), and 3-methoxytyramine (3-MT) were displayed as double derivatized ions with FMP-10. Homovanillic acid (HVA) was displayed as single derivatized ion. Ion intensities are shown using a rainbow color coded linear scale from 0 to 100% (HVA is displayed with a scale from 0 to 50%). Abbreviations: CPu, caudate putamen; NAc, nucleus accumbens; AcbC, accumbens nucleus, core; AcnSh, accumbens nucleus, shell; LS, lateral septum; SHi, septohippocampal nucleus; cc, corpus callosum; aca, anterior commissure; LV, lateral; AD, aldehyde dehydrogenase.

References

1. Shariatgorji M, Nilsson A, Fridjonsdottir E, Vallianatou T, Källback P, Katan L *et al.* Comprehensive mapping of neurotransmitter networks by MALDI–MS imaging. *Nature Methods* 2019; **16**(10)**:** 1021-1028.

2. Hertel P, Nomikos GG, Iurlo M, Svensson TH. Risperidone: regional effects in vivo on release and metabolism of dopamine and serotonin in the rat brain. *Psychopharmacology* 1996; **124**(1)**:** 74-86.
